# Dual regulation of NEMO by Nrf2 and miR-125a inhibits ferroptosis and protects liver from endoplasmic reticulum stress-induced injury

**DOI:** 10.7150/thno.89703

**Published:** 2024-02-24

**Authors:** Jihoon Tak, Min Sung Joo, Yun Seok Kim, Hyun Woo Park, Chang Hoon Lee, Gil-Chun Park, Shin Hwang, Sang Geon Kim

**Affiliations:** 1 College of Pharmacy, Seoul National University, Seoul 08826, Republic of Korea.; 2 Department of Clinical Pharmacology and Therapeutics, Seoul National University College of Medicine, Seoul 03080, Republic of Korea.; 3 Department of Surgery, Asan Medical Center, University of Ulsan College of Medicine, Seoul, Republic of Korea.; 4 College of Pharmacy and Integrated Research Institute for Drug Development, Dongguk University-Seoul, Goyang-si, Kyeonggi-do 10326, Republic of Korea.

**Keywords:** NEMO, Nrf2, miR-125a, Acute liver injury, Ferroptosis

## Abstract

**Rationale:** The surge of severe liver damage underscores the necessity for identifying new targets and therapeutic agents. Endoplasmic reticulum (ER) stress induces ferroptosis with Gα_12_ overexpression. NF-κB essential modulator (NEMO) is a regulator of inflammation and necroptosis. Nonetheless, the regulatory basis of NEMO *de novo* synthesis and its impact on hepatocyte ferroptosis need to be established. This study investigated whether Nrf2 transcriptionally induces *IKBKG* (the NEMO gene) for ferroptosis inhibition and, if so, how NEMO induction protects hepatocytes against ER stress-induced ferroptosis.

**Methods:** Experiments were conducted using human liver tissues, hepatocytes, and injury models, incorporating NEMO overexpression and Gα_12_ gene modulations. RNA sequencing, immunoblotting, immunohistochemistry, reporter assays, and mutation analyses were done.

**Results:** NEMO downregulation connects closely to ER and oxidative stress, worsening liver damage via hepatocyte ferroptosis. NEMO overexpression protects hepatocytes from ferroptosis by promoting glutathione peroxidase 4 (GPX4) expression. This protective role extends to oxidative and ER stress. Similar shifts occur in nuclear factor erythroid-2-related factor-2 (Nrf2) expression alongside NEMO changes. Nrf2 is newly identified as an *IKBKG* (NEMO gene) transactivator. Gα_12_ changes, apart from Nrf2, impact NEMO expression, pointing to post-transcriptional control. Gα_12_ reduction lowers miR-125a, an inhibitor of NEMO, while overexpression has the opposite effect. NEMO also counters ER stress, which triggers Gα_12_ overexpression. Gα_12_'s significance in NEMO-dependent hepatocyte survival is confirmed via ROCK1 inhibition, a Gα_12_ downstream kinase, and miR-125a. The verified alterations or associations within the targeted entities are validated in human liver specimens and datasets originating from livers subjected to exposure to other injurious agents.

**Conclusions:** Hepatic injury prompted by ER stress leads to the suppression of NEMO, thereby facilitating ferroptosis through the inhibition of GPX4. *IKBKG* is transactivated by Nrf2 against Gα_12_ overexpression responsible for the increase of miR-125a, an unprecedented NEMO inhibitor, resulting in GPX4 induction. Accordingly, the induction of NEMO mitigates ferroptotic liver injury.

## Introduction

The emergence of acute liver injury (ALI) presents a clinical challenge and represents a key focal point within clinical practice and public health apprehensions 1, 2. It encompasses hepatocellular impairment induced by pharmaceutical agents, herbal compounds, or other chemical entities. This spectrum extends from subtle, asymptomatic elevations in hepatic enzymes to profound hepatotoxicity, ultimately leading to acute hepatic failure or requiring transplantation 3. Among many drugs and chemicals implicated in ALI, acetaminophen (APAP) intoxication has been a prominent cause 4, 5. So, APAP-induced hepatotoxicity draws substantial public attention, prompting research to ascertain its pathology and underlying mechanisms. Nonetheless, the molecular basis of APAP-induced ALI remains incomplete.

Ferroptosis is a form of regulated cell death characterized by lipid peroxide accumulation and oxidative damage, which are the key features associated with APAP-induced liver injury. This liver injury also occurs when glutathione peroxidase 4 (GPX4) is suppressed in the cell, which leads to a decrease in reduced glutathione (GSH) content and impairs cellular defense against lipid peroxidation, exacerbating ferroptosis 6-9. This type of cell death also relies on intracellular iron contents 10. Moreover, ferroptosis is closely linked with endoplasmic reticulum (ER) stress, one of the mechanisms implicated in liver pathophysiology 11, 12. Sustaining ER stress indeed enhances ferroptosis by promoting iron overload through autophagy 13. Despite the link between ferroptosis and ER stress, the molecular association of ferroptosis and ER stress regulation should be characterized in the context of ALI progression.

The IκB kinase (IKK) complex, comprising subunits IKKα, IKKβ, and IKKγ (also called NF-κB essential modulator, NEMO), is a vital inflammation and cell death regulator 14. Virus infection, ionizing radiation, TNFα-receptor activation, and other external stimuli form the IKKα/β/γ complex, inducing inflammatory cytokines and pathogen-associated molecular patterns. Of these isoforms, NEMO forms a high molecular complex with IKKα and IKKβ for IκBα degradation and NF-κB activation 15-17. Decreased NEMO levels engender necroptosis through RIPK1/RIPK3. Thus, liver-specific NEMO knockout enhances liver injury 18, 19. Similarly, NEMO knockout in the colon prompts epithelial cell death, facilitating inflammatory colon disease 20. Thus, the pathogenesis of APAP-induced ferroptosis needs to be elucidated in conjunction with NEMO dysregulation.

Nuclear factor erythroid-2-related factor2 (Nrf2) protects cells from oxidative injury triggered by external stresses, including xenobiotic intoxication, through antioxidant enzyme induction and inflammatory stress inhibition 21-23. Nrf2 also controls cell fate determination, induces cell survival molecules such as Bcl-2 and certain microRNAs (e.g., miR-125b), and inhibits pro-apoptotic molecules 24, 25. In addition to apoptosis, Nrf2 activation attenuates necrosis or pyroptosis 26, 27. Since the regulatory roles of Nrf2 in ER stress-induced ferroptosis and its associated targets remain elusive, the present study sought to explore antioxidant proteins that can be transcriptionally controlled by Nrf2 as part of an effort to find the regulator(s). This study investigated whether Nrf2 transcriptionally induces *IKBKG* (the NEMO gene) for ferroptosis inhibition and, if so, how NEMO induction protects hepatocytes against ER stress-induced ferroptosis. These findings reveal Nrf2's previously unrecognized role as a transcriptional *IKBKG* regulator, providing insights into the underlying mechanisms that govern ferroptosis via GPX4.

In our recent investigation, APAP-induced liver injury was characterized by ferroptosis via GPX4 from ER stress-mediated Gα_12_ overexpression 28. These findings evidence a potential ferroptosis involvement in APAP-induced liver injury pathogenesis and highlight the need for further investigation into the APAP toxicity and ferroptosis relationship. Another objective of the present study was to ascertain whether Gα_12_ signaling regulates the Nrf2-NEMO axis, potentially influencing ferroptosis under APAP toxicity. Thus, apart from Nrf2's induction of *IKBKG*, we wondered if NEMO expression is under the control of the Gα_12_ signaling pathway. In this inquiry, this study intriguingly identified miR-125a as a newly determined NEMO inhibitor downstream of the Gα_12_-ROCK1 axis. Consequently, our research results support the dual regulatory pathways of NEMO expression by Nrf2 and miR-125a, which contributes to the inhibition of ferroptosis, protecting the liver from ER stress-induced ALI. The outcomes were validated through patient specimen examination, consolidating identified target relevance in human liver pathophysiology. Hence, this research may offer insights into discovering potential approaches for the treatment of ALI.

## Materials and methods

### Human sample analysis

Human liver specimens were obtained from donors and recipients undergoing liver transplantation from 2011 to 2020 after histologic examination and ultrasonography at Asan Medical Center (Seoul, South Korea) for ALI analysis. During procurement, an intraoperative liver assessment was systematically performed to rule out fibrosis, cirrhosis, steatosis, and other abnormalities before transplantation. All patients in this study provided written informed consent. This study was approved by Asan Medical Center's Institutional Review Board (IRB no. 2021-0839) and adhered to the 1975 Declaration of Helsinki ethical guidelines.

### Animal models and experiments

The animal experiment protocol was approved by the Seoul National University's Institutional Review Board and conducted under the Institutional Animal Care and Use Committee (IACUC) at Seoul National University (No. SNU-171115-2, SNU-200723-2-2, SNU-201229-4, SNU-201229-5, SNU-201229-6) guidelines. The mice were housed in a 12 h light/dark cycle and relative humidity of 50% ± 5% under filtered, pathogen-free air, with food and water available *ad libitum*. Male mice at 8 to 12 weeks of age, unless otherwise indicated, were used. To mitigate environmental disparities, mice were acclimated for a minimum of one week preceding each experimental procedure. For the establishment of an acute liver injury model, male C57BL/6 mice underwent overnight fasting followed by a single intraperitoneal dose of APAP treatment (300 mg/kg BW, i.p.), and the liver and blood samples were collected 6 h afterward; In this experiment, we chose a 6 h time point because APAP treatment at the above dose increased serum ALT activities up to 24 h, which peaked 6 h post-treatment 29. Where indicated, mice were given an injection of ferrostatin-1 (1 mg/kg BW, i.p.) 1 h prior to APAP treatment.

Separately, carbon tetrachloride (CCl_4_, 0.5 mL/kg BW, 1:20 in corn oil, i.p.) was administered twice weekly over the specified duration (6 weeks) as a liver injury model. For the induction of ER stress, male C57BL/6 mice were received a single intraperitoneal injection of 2 mg/kg tunicamycin (Tm, Sigma Aldrich) in 150 mM dextrose for 72 h, whereas only dextrose solution was injected into control mice. Male C57BL/6 mice underwent overnight fasting before a single dose of BSO treatment (1 g/kg BW, i.p.), and tissue and blood samples were obtained 6 h later. In the context of an *in vivo* rescue experiment, male C57BL/6 mice were fasted overnight and treated with a single dose of APAP (300 mg/kg BW, i.p.). After 1 h, the mice were subjected to ripasudil exposure (50 mg/kg BW, i.p.), and tissue samples were collected 5 h afterward.

* Nrf2* knockout (KO) mice supplied by RIKEN BioResource Center (Tsukuba, Japan) were bred and maintained. Details of the generation of the *Gna12* KO mice used in this study have been described previously 30.

### Statistical analyses

Statistical significance was tested via two-tailed Student's t-tests, Mann-Whitney U test, one-way ANOVA coupled with Bonferroni's method, Tukey's honestly significant difference test, or the least significant difference multiple comparison procedure, when appropriate. Correlation coefficients (r) were determined via Pearson's or Spearman's correlation methods. Differences were considered significant at *P* < 0.05. Statistical analyses were performed using IBM SPSS Statistics 26 software or Prism version 8.0 (GraphPad Software).

Additional details regarding materials and experimental protocols are provided in the Supplementary Materials and Methods.

## Results

### Inhibition of *IKBKG* transcript levels in liver injury patients

**Figure 1:**
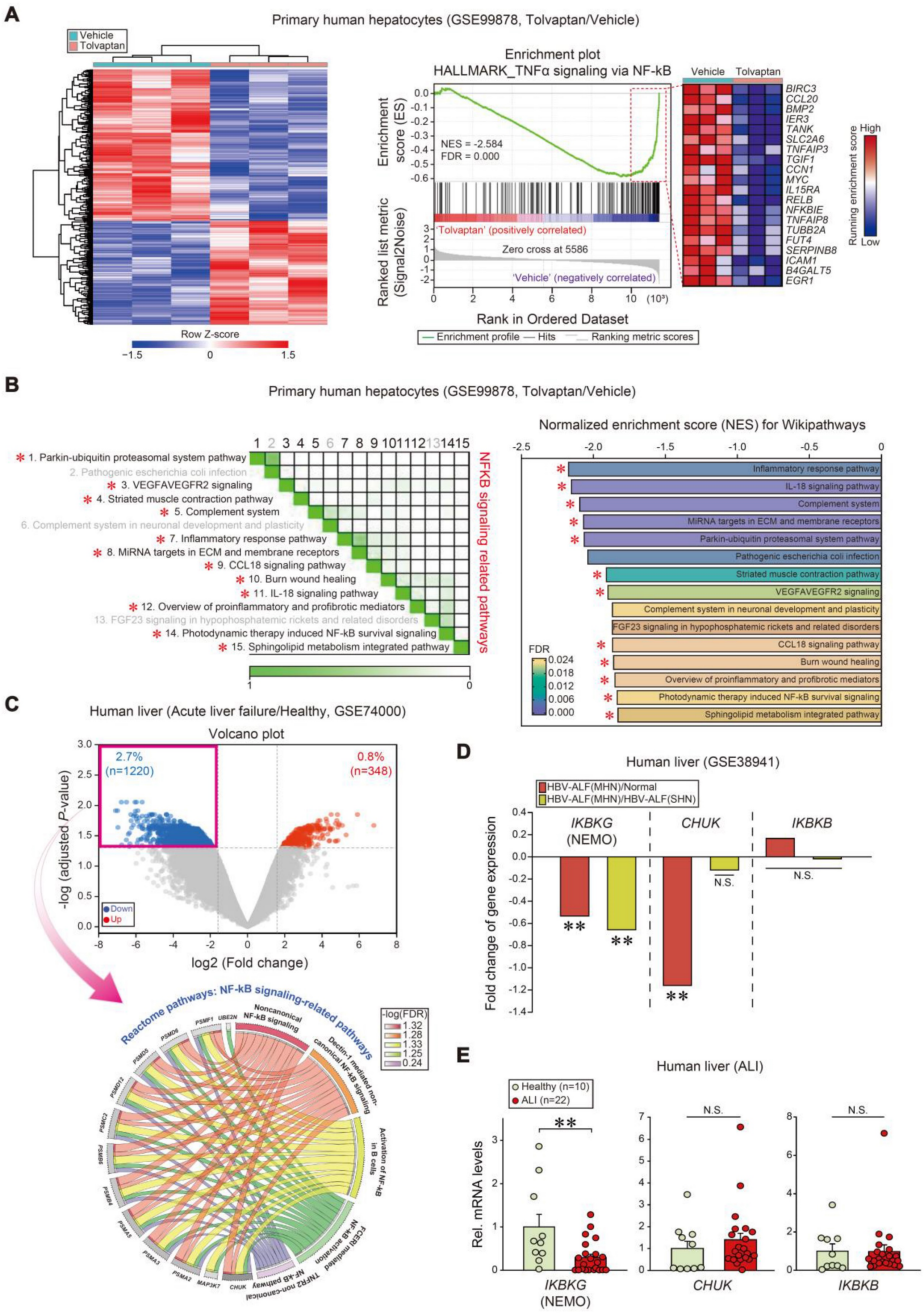
** Inhibition of *IKBKG* transcript levels in ALI patients. (A)** Heatmap and hierarchical correlation analysis of DEGs (absolute fold-change > 1.5 and *P* < 0.05) (left) and GSEA-enrichment plot of representative gene sets (NES = -2.584, FDR = 0.000) negatively correlated with tolvaptan treatment in primary human hepatocytes using hepatic transcriptome data (n = 3 each, GSE99878) (right). The top 20 genes comprising the enrichment score's leading edge are indicated in the corresponding heatmap (blue, low; red, high). **(B)** Leading-edge analysis (left) and bar graphs (right) of significantly enriched GSEA Wikipathways using the same data as in **A**. GSEA leading-edge analysis results are represented as a matrix where the green color intensity indicates the overlap degree between core genes in each gene set combination; the more intense the green color, the greater the overlap. NES and FDR are presented as a bar graph (NES < -1.82, FDR < 0.024). NF-κB signaling-related pathways were marked with red asterisks. **(C)** Volcano plot (upper) of RNA-seq data from a public dataset (GSE74000, n = 2 or 3 each). Horizontal and vertical lines indicate the filtering criteria (absolute fold-change > 1.5 and adjusted *P* < 0.05, respectively). Red and blue dots indicate upregulated or downregulated differentially expressed genes (DEGs) in acute liver failure patients. Reactome pathway analysis of DEGs identified NF-κB-related pathway enrichment in acute liver failure patients (lower). The Circos plots illustrate the enriched DEGs overlaps and specific responses in significant Reactome pathways. Hallmark genes and associated pathways are color-coded and are represented by a specific color in the inner ring. The ribbon/arc that originates from different genes and terminates at associated Reactome pathways demonstrates the connectivity between genes and Reactome pathways. **(D)** Fold change of IkappaB kinase (IKK) complex transcript levels in livers of healthy individuals or HBV-acute liver failure (ALF) patients. MHN, massive hepatic necrosis; SHN, submassive hepatic necrosis (n = 10 samples from 10 individual normal subjects; n = 8 samples from 2 HBV-ALF(SHN) patients; n = 9 samples from 2 HBV-ALF(MHN) patients, as described in the GSE38941 database). **(E)** *IKBKG*, *CHUK*, and *IKBKB* transcript levels in livers of healthy individuals (n = 10) or ALI patients (n = 22). For **E**, values were expressed as mean ± SEM (*P < 0.05, **P < 0.01). Statistical significance was tested via two-tailed Mann-Whitney U test.

As a first step toward identifying prominent liver injury regulators, pathways were analyzed using a public dataset (GSE99878); Tolvaptan, known for its capacity to cause ALI, induced a marked shift in the transcriptional heatmap profile (Figure 1A, left). Notably, NF-κB signaling process-associated gene sets were the most downregulated (first rank) in tolvaptan-treated human primary hepatocytes among hallmark pathways (Figure 1A, right). In addition, NF-κB-related gene sets were diminished among the top 15 Wikipathways in subsequent leading-edge analyses, which elucidated overlaps between NF-κB-related pathways and the associated genes (Figure 1B).

Next, we assessed gene ontology pathway changes in APAP-induced acute liver failure patients and healthy individuals (GSE74000); 1220 genes were significantly downregulated, whereas 348 genes were upregulated (Figure 1C, upper). Interestingly, the genes were tagged by several gene ontology (GO) terms analyzed in the Reactome pathway; 13 of each were clustered into five GO groups, belonging to the 'NF-κB signaling-related pathways' among downregulated genes in patients (Figure 1C, lower). GO and biological process analyses of the same dataset confirmed downregulated genes associated with the NF-κB pathway in the same patients (Figure S1A). Similarly, GO and biological process analyses indicated enhanced 'regulation of acute inflammatory response' gene expressions (Figure S1B).

To explore the new functional molecule(s), we focused on the IκB kinase (IKK) that participates in upstream NF-κB signaling and promotes NF-κB activation through pro-inflammatory stimuli 31.

In another public GEO database analysis regarding HBV-associated acute liver failure (HBV-ALF) patients (GSE38941), we assessed fold change values obtained from mRNA levels of enzyme complex IKK subunits (i.e., IKKα, IKKβ, and IKKγ). Relative levels in mRNA expression of *IKBKG* and *CHUK* were significantly downregulated in patients with hepatic necrosis, whereas *IKBKB* was unchanged (Figure 1D). To validate the *IKBKG* and *CHUK* association, we used our own samples of patients with ALI on drugs (i.e., liver intoxications from herbs, drug medications, or unknown origins), as described previously 28. Among transcripts, *IKBKG* was the most significantly diminished in patients with ALI compared to healthy subjects (i.e., -70.7%) (Figure 1E). These results substantiate the inverse *IKBKG* transcript level and hepatic injury association.

### Nrf2 and NEMO associations during liver injury

**Figure 2:**
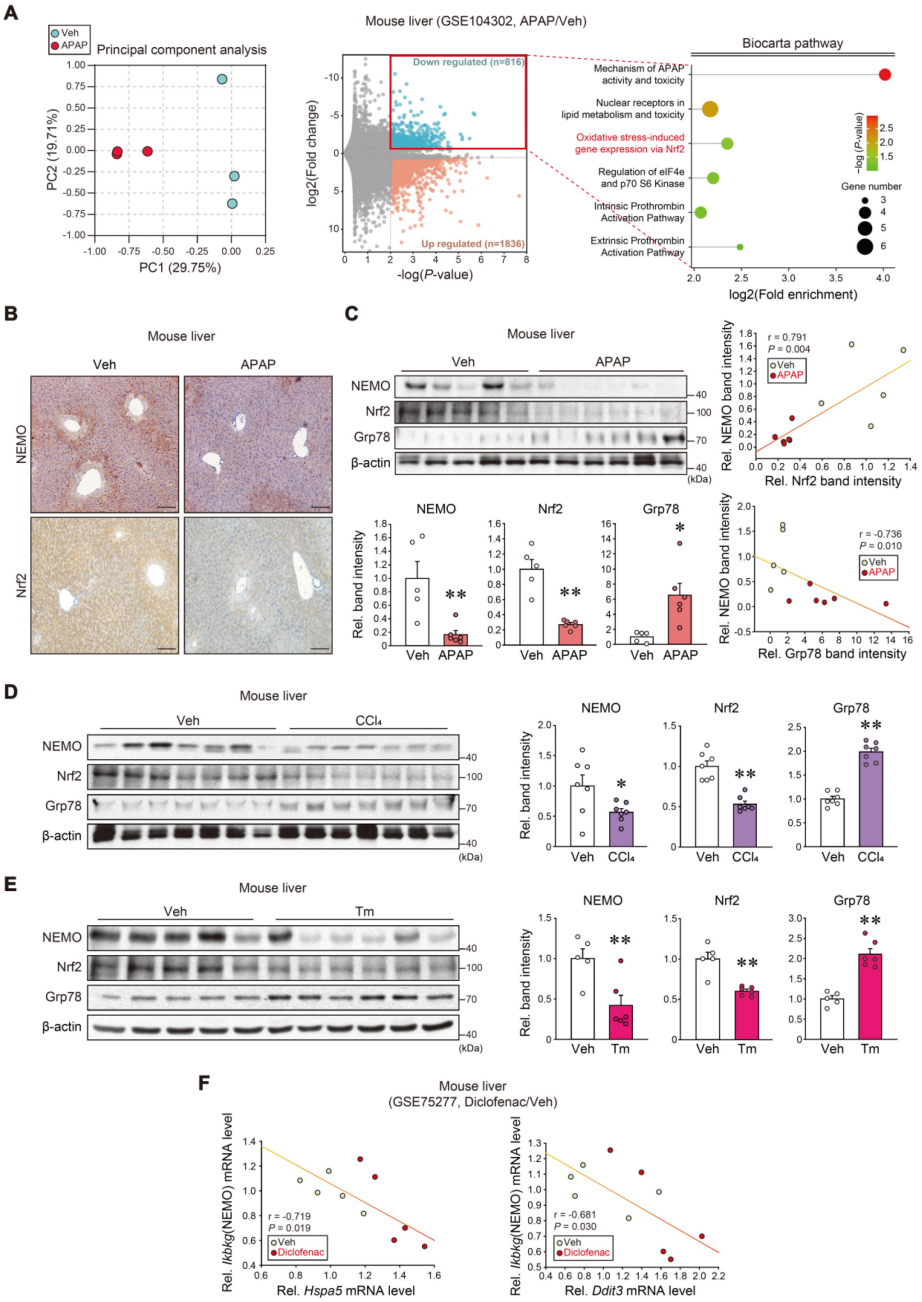
** The Nrf2 and NEMO relationship and association with ER stress responses. (A)** RNA-seq dataset (GSE104302) obtained from the liver of APAP- or vehicle-treated mice. Principal component analysis (PCA) score (left) and volcano plots of RNA-seq data (middle) (mint color, downregulated; red color, upregulated; DEGs with *P*-value < 0.01 and absolute FC > 1.5). Enrichment bubble plot of the Biocarta pathway indicating that the 'Oxidative stress-induced gene expression via Nrf2' was significantly downregulated in response to APAP treatment (n = 3 each) (right). **(B)** NEMO and Nrf2 immunohistochemistry in the liver of the mice treated with a single APAP dose (300 mg/kg BW, i.p., 6 h) (n = 5 or 6 each). **(C)** NEMO, Nrf2, and Grp78 immunoblottings (upper left) in the livers of the same mice as in **B**. Band intensities represent values relative to each respective control (n = 5 or 6 each) (lower left). Nrf2 and NEMO correlations (upper right); Grp78 and NEMO correlations (n = 11) (lower right). **(D, E)** NEMO, Nrf2, and Grp78 immunoblottings (left) in the liver of CCl_4_-treated mice (0.5 mL/kg BW, i.p., 6 weeks) **(D)** or those treated with a single Tm dose (2 mg/kg BW, i.p., 72 h) **(E)**. Band intensities represent values relative to each respective control (n = 7 each for **D**; and n = 5 or 6 each for **E**) (right). **(F)** ER stress markers (*Hspa5*, *Ddit3*) and *Ikbkg* transcript correlations using a public domain GSE75277 dataset concerning the liver of diclofenac-treated mice (30 mg/kg BW, i.p., 14 days) or the vehicle (n = 5 each). For **C-E**, values were expressed as mean ± SEM (*P < 0.05, **P < 0.01). Statistical significance was tested via two-tailed Student's *t*-tests and Pearson's correlation analyses.

We first analyzed the RNA sequencing (RNA-seq) dataset from APAP-subjected mice (GSE104302) to explore NEMO regulation. Principal component analysis (PCA) exhibited a gene expression segregation between APAP and the vehicle (Figure 2A, left). Differentially expressed genes (DEGs) accounted for 11.5% of the entire transcriptomes; Among the 2652 DEGs, 816 were downregulated, whereas 1836 were upregulated (Figure 2A, middle). In the Biocarta analysis for the downregulated gene group, several biological processes expressed high-fold enrichments in 'Mechanism of APAP activity and toxicity' and 'Extrinsic prothrombin activation' (foremost affected), and 'Oxidative stress-induced gene expression via Nrf2' (second-most affected) (Figure 2A, right). Furthermore, we analyzed GO gene sets and GSEA hallmarks using the transcriptome dataset from mouse liver (GSE104302, GSE173595); APAP treatment affected protein refolding genes (Figure S2A) and those linked to the 'unfolded protein response' (Figure S2B).

Considering the Nrf2 and hepatic injury association, we experimentally assessed the potential Nrf2 and NEMO correlation. APAP treatment in mice inhibited NEMO and Nrf2 in the liver with increased Grp78 levels (Figure 2B and C), as confirmed in primary hepatocytes (Figure S2C). Additionally, we examined other liver injury inducer effects (i.e., carbon tetrachloride [CCl_4_] and tunicamycin [Tm]), discovering that the toxicants treatment displayed similar effects (Figure 2D and E). In analyzing the diclofenac (i.e., an agent that causes liver injury) medication dataset, *Ikbkg* mRNA levels were inversely correlated with those of cellular stress markers (*Hspa5* and *Ddit3*) (Figure 2F). These findings support Nrf2 and NEMO inhibition and their correlations with liver injury.

### NEMO induction of GPX4 for the inhibition of ferroptosis

**Figure 3:**
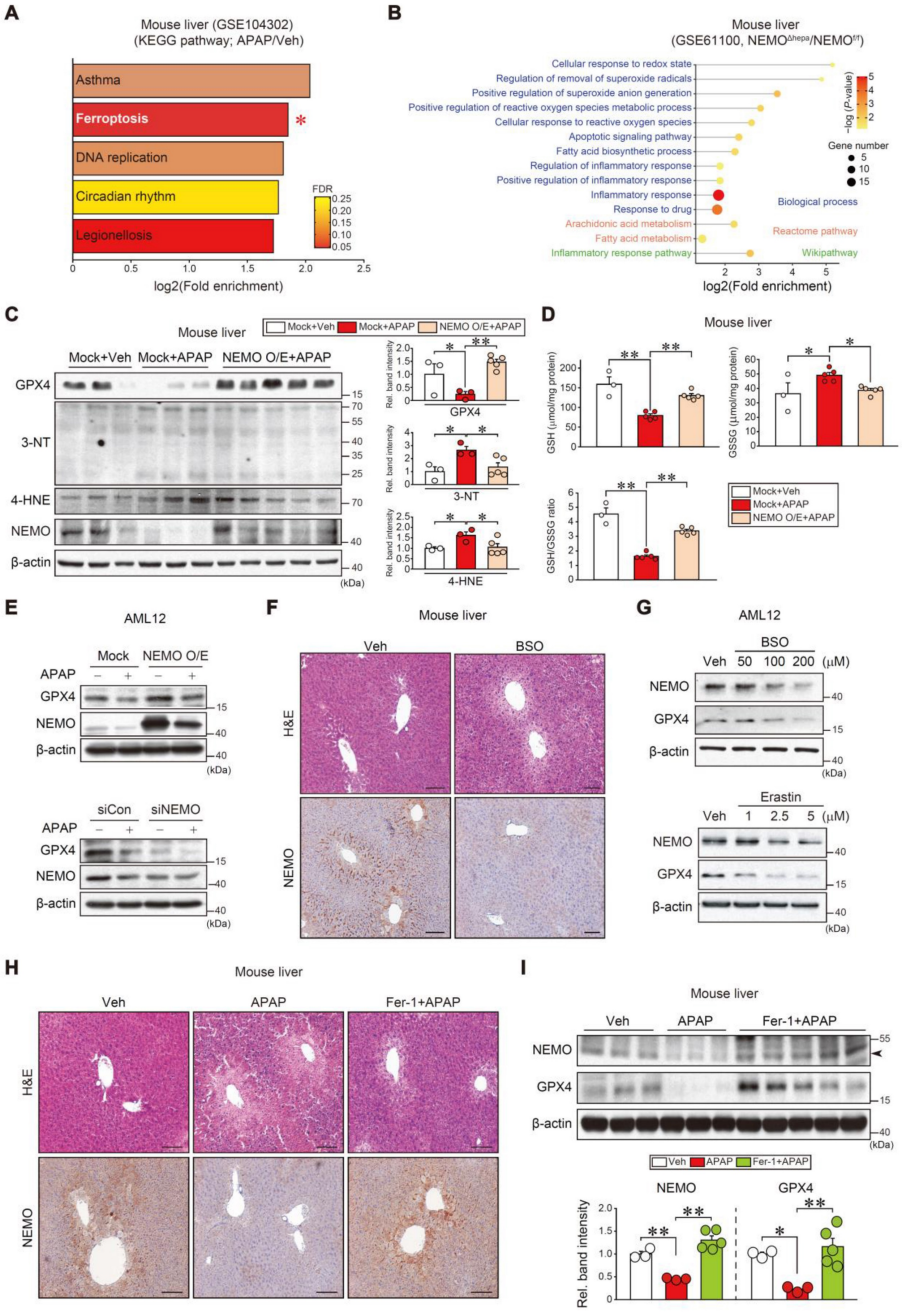
** Toxicant-induced ferroptosis inhibition through NEMO. (A)** KEGG analysis using a cDNA microarray dataset obtained from WT mice livers treated with APAP or the vehicle. The ferroptosis pathway (red asterisk) exhibited high fold enrichment (second rank) (n = 3 each, DEGs of *P*-value < 0.01 and FC > 2, GSE104302). FDR is indicated in the bar graph. **(B)** Ferroptosis-related pathways based in Biological process, Reactome pathway, and Wikipathway obtained from NEMO^f/f^ or NEMO^Δhepa^ mice livers (n = 3 each, DEGs of *P*-value < 0.05 and FC > 2, GSE61100). **(C)** Immunoblottings for representative ferroptosis markers in WT mice livers treated with a single APAP dose (300 mg/kg BW, i.p., 6 h) 3 days post-hydrodynamic injection with NEMO or control plasmid DNA (25 μg each) via tail vein (left). Band intensities represent values relative to each respective control (n = 3 or 5 each) (right). **(D)** Reduced glutathione (GSH), oxidized glutathione disulfide (GSSG), and GSH/GSSG ratio measurements in WT mice livers treated with a single APAP dose (300 mg/kg BW, i.p., 6 h) 3 days post-hydrodynamic injection with NEMO or control plasmid DNA (25 μg each) via tail vein (n = 3 or 5 each). **(E)** GPX4 and NEMO immunoblottings for APAP-treated AML12 cells (10 mM, 12 h) after transfection with NEMO (Mock, 1 µg, 24 h) (upper) or siNEMO (siCon, 100 nM, 24 h) (lower) (n = 3; repeated three times with similar results). **(F)** Liver histopathology and immunohistochemistry for NEMO in the livers of mice treated with a single dose of BSO (1 g/kg BW, i.p., 6 h) (n = 3 each). Scale bar, 200 µm. **(G)** Immunoblottings for NEMO and GPX4 in AML12 cells treated with the indicated BSO (upper) and Erastin (lower) concentrations for 12 h (n = 3; repeated three times with similar results). **(H)** Liver histopathology and immunohistochemistry for NEMO in the livers of mice treated with APAP (300 mg/kg BW, i.p., 6 h) 1 h after vehicle or Fer-1 (1 mg/kg BW, i.p.) treatment. Scale bar, 200 µm. **(I)** Immunoblottings for NEMO and GPX4 in the same mice as in **H** (upper). Band intensities represent values relative to the respective control (n = 3 or 5 each) (lower). For **C, D**, and **I** values were expressed as mean ± SEM (*P < 0.05, **P < 0.01). Statistical significance was tested via one-way ANOVA coupled with Bonferroni's method or the LSD multiple comparison procedure when appropriate.

To understand the direct effect of chemical intoxication on NEMO's uncharacterized role in a different hepatocyte death pathway, we performed the KEGG analysis using our dataset (GSE104302), APAP intoxication promoted the ferroptosis pathway (second rank) among the different types of cell death (Figure 3A and Figure S3A). Moreover, NEMO ablation in hepatocytes upregulated ferroptosis-associated gene sets in other Biological processes, Reactome pathways, and Wikipathway analyses (Figure 3B). In subsequent experiments, NEMO's modulation of ferroptosis biomarkers in the liver and hepatocytes were examined. NEMO overexpression in hepatocytes, through hydrodynamic injection in mice 32, completely reversed the inhibitory effect of APAP on GPX4 along with decreases in 3-nitrotyrosine (3-NT) and 4-hydroxynonenal (4-HNE) levels (Figure 3C). Consistently, the reduced glutathione (GSH) content was recovered in the liver (Figure 3D). In AML12 cells, NEMO overexpression and siRNA knockdown affected GPX4 levels (Figure 3E), corroborating NEMO's direct antioxidant effect in hepatocytes. In addition, APAP treatment reduced *Gpx4* transcript levels, which was not reversed by NEMO overexpression (Figure S3B), suggesting that NEMO is unlikely to transcriptionally activate GPX4. In the cycloheximide experiment, modulations of NEMO by overexpression and siRNA knockdown changed GPX4 levels, supportive of NEMO's effect on GPX4 stabilization (Figure S3C). Moreover, NEMO overexpression prevented APAP from increasing Fe^2+^ levels (Figure S3D). We further examined mitochondrial fusion and fission marker transcripts (i.e., *Mfn1*, *Mfn2*, and *Opa1* for fusion; and *Fis1*, and *Drp1* for fission); APAP treatment decreased all of the marker transcripts, but this effect was not changed by NEMO overexpression (Figure S3E).

Subsequently, we confirmed that treatment with DL-buthionine-[S, R]-sulfoximine (BSO), an agent that induces ferroptosis and depletes cellular GSH through GPX4 inhibition 10, inhibited NEMO in the liver and AML12 cells. Erastin, another ferroptosis inducer, also inhibited NEMO and GPX4 (Figure 3F and G). Consistently, treatment with ferrostatin-1 (Fer-1), a specific antioxidative ferroptosis inhibitor 6, 33, ameliorated liver injury and enhanced NEMO and GPX4 levels against APAP (Figure 3H and I). Similar outcomes were obtained for Nrf2, NEMO, and GPX4 in mouse primary hepatocytes (Figure S3F), supporting the antioxidant spin-trapping effect of Fer-1. In this event, an increase of Nrf2 by Fer-1 may contribute to GPX4 expression against APAP. All of these results provide evidence that NEMO inhibits hepatic ferroptosis by upregulating GPX4 and is controlled by ferroptosis inducers and inhibitors in APAP-induced ALI.

### Protective NEMO effects against oxidative- and ER stress-induced hepatic injury

**Figure 4:**
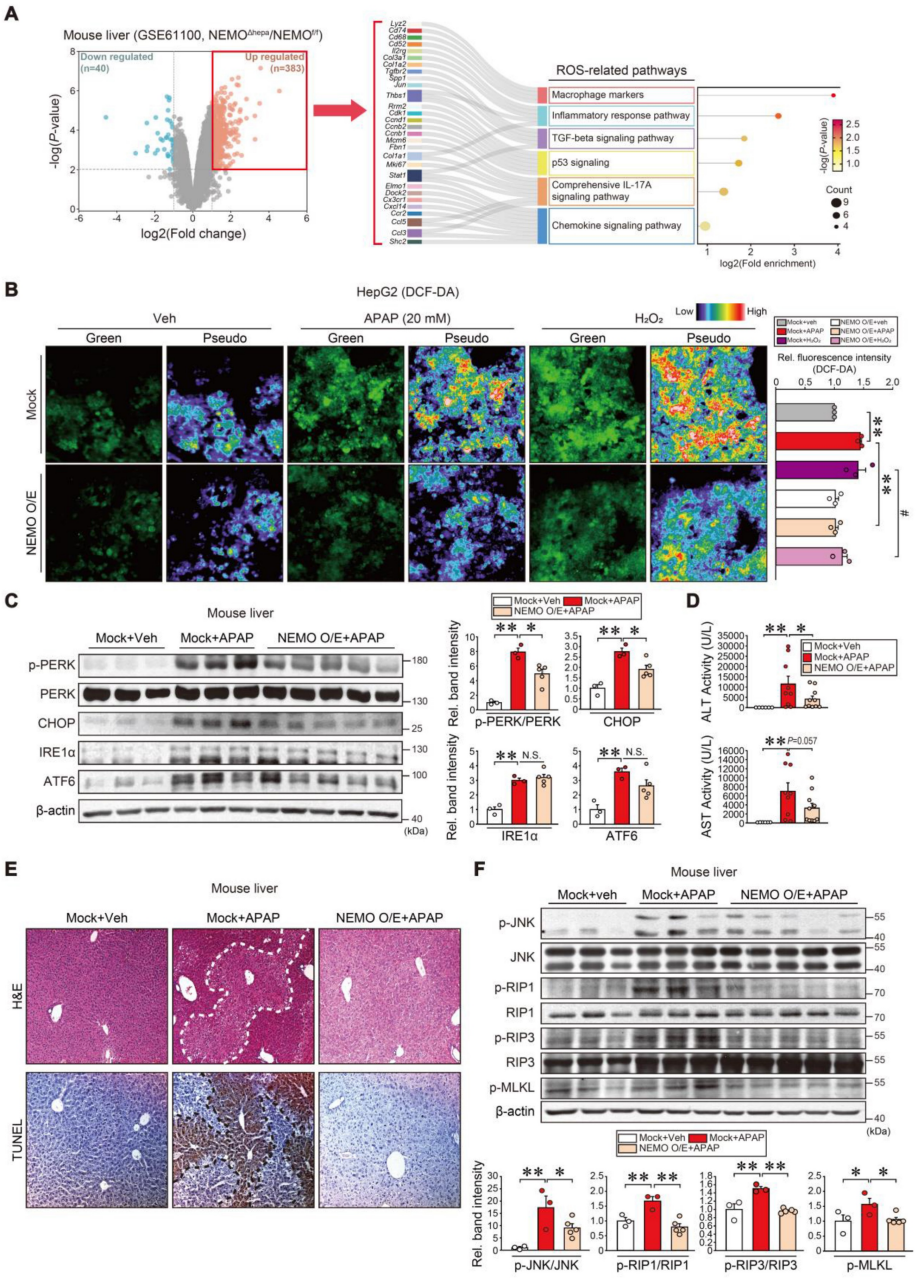
** Inhibition of toxicant-induced liver injury through NEMO. (A)** Volcano plot (left) (mint, downregulation; red, upregulation) and ROS-related pathways based on the Sankey diagram (plot) of DEGs using hepatic transcriptome data from NEMO^f/f^ or NEMO^Δhepa^ mice (right). The Sankey diagram represents genes within each pathway; dot plots with sizes indicate gene numbers and dot colors display *P*-values (n = 3 each, DEGs of *P*-value < 0.01 and FC ≥ 2, FC ≤ -2, GSE61100). **(B)** Representative fluorescence images of DCF-DA (2′,7′-dichlorofluorescein diacetate) staining for ROS detection in HepG2 cells treated with APAP (20 mM, 12 h) or H_2_O_2_ (100 μM, 10 min) as a positive control after NEMO transfection (or Mock) (1 µg, 24h) (left). Green and pseudocolor images were captured, and the relative color scale indicates DCF fluorescence levels. Scale bar, 100 μm. DCF-DA-enhanced fluorescence-positive cells were analyzed with Image J software (right). **(C)** Immunoblottings for representative ER stress markers in the same samples as in **Fig. 3C** (left). Band intensities represent values relative to each respective control (n = 3 or 5 each) (right). **(D)** Serum alanine transaminase (ALT) and aspartate transaminase (AST) activities in WT mice livers treated with a single APAP dose (300 mg/kg BW, i.p., 6 h) 3 days post-hydrodynamic injection with NEMO or control plasmid DNA (25 μg each) via tail vein (n = 6-10 each). **(E)** Liver histopathology (H&E) and terminal deoxynucleotidyl transferase dUTP nick-end labeling (TUNEL) assays. H&E and TUNEL stainings were done on the same mice livers as in **Fig. 3C**. Scale bar, 200 µm. **(F)** Immunoblottings for representative necroptosis markers in the same samples as in **Fig. 3C** (upper). Band intensities represent values relative to each respective control (n = 3 or 5 each) (lower). For **B-D** and **F**, values were expressed as mean ± SEM (*P < 0.05, **P < 0.01). Statistical significance was tested via one-way ANOVA coupled with Bonferroni's method or the LSD multiple comparison procedure when appropriate.

In subsequent studies, we examined NEMO effects on oxidative stress and ER stress in hepatocytes. In the public RNA-seq dataset analysis (GSE61100), NEMO gene knockout resulted in DEGs accounting for 1.7% of total transcriptomes; Among the 423 DEGs, 40 were downregulated, while 383 were upregulated (Figure 4A, left). NEMO abrogation upregulated 383 genes, of which 29 were associated with reactive oxygen species (ROS) pathways in the Sankey diagram visualization (i.e., macrophage markers and inflammatory response pathways) (Figure 4A, right). Conversely, downregulated genes were related to lipid metabolic processes in the dataset from hepatocyte-specific NEMO knockout mice (Figure S4A). Hallmark pathway analysis of our RNA-seq dataset (GSE173595) from APAP-treated mice conveyed upregulated gene sets associated with ROS and ER stress (Figure S4B and C) 34. In our experiment, NEMO overexpression prevented APAP and H_2_O_2_ from increasing DCF fluorescent intensity in HepG2 cells (Figure 4B), confirming NEMO's ability to inhibit ROS. In addition, NEMO overexpression in hepatocytes through hydrodynamic gene deliveries attenuated p-PERK and CHOP intensities increased by APAP treatment (Figure 4C). IRE1α, ATF6, and other stress markers were largely unaffected. In addition, PERK overexpression enhanced APAP's inhibition on GPX4, whereas PERK siRNA exerted the opposite effect; However, PERK modulations did not change NEMO levels (Figure S5). These data support the notion that NEMO increases GPX4 by inhibiting PERK against ER stress. Furthermore, NEMO overexpression inhibited ALT and AST activities, ameliorating liver histopathology and TUNEL staining intensity (Figure 4D and E). Consistently, cell death markers including p-JNK, p-RIP1, p-RIP3, and p-MLKL were all inhibited (Figure 4F). Our results support the concept that NEMO inhibits oxidative stress and ER stress in association with liver protection.

### Nrf2 as a transcriptional *IKBKG* gene regulator

**Figure 5:**
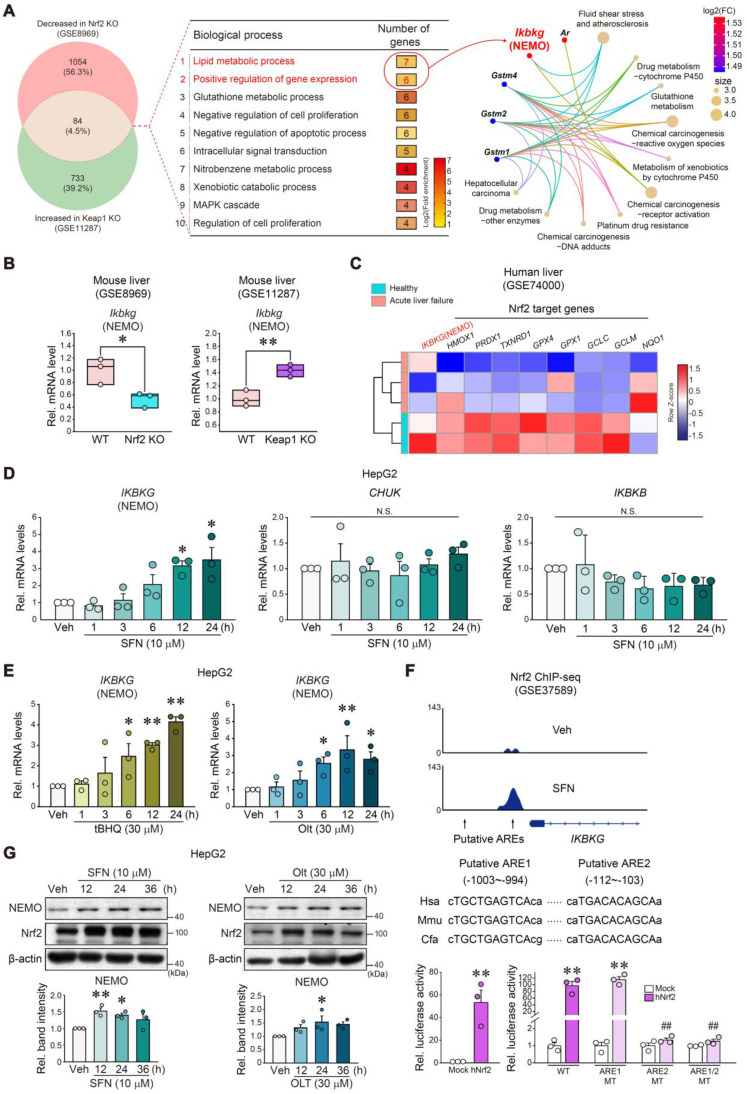
** Nrf2-mediated *IKBKG* (NEMO) transactivation. (A)** Gene Venn diagrams of downregulated and upregulated genes in liver samples from Nrf2 and Keap1 knockout mouse models (n = 3 each, GSE8969 and GSE11287; DEGs of *P*-value < 0.05 and absolute FC > 1.5, respectively) (left). GO term enrichment of overlapped genes (84 genes, 4.5%) between two groups for biological processes, such as the lipid metabolic process (first rank) and positive gene expression regulation (second rank) (middle). The gene-concept network (cnetplot) of functional GO enrichment results (right) from the leading genes highlighted in the circles' red areas in the first and second biological process ranks. The cnetplot depicts gene and biological concept (GO terms) linkages as a network. Circle size indicates genes represented in a given biological process. **(B)** *Ikbkg* transcript levels obtained using a public dataset concerning livers of WT and Nrf2 KO (GSE8969) or Keap1 KO mice (GSE11287). **(C)** Heatmap of significantly down-regulated *IKBKG* transcript and Nrf2 target genes in APAP-intoxicated patients (n = 2 or 3, GSE74000). **(D, E)** Real-time RT-PCR assays for *IKBKG, CHUK,* and* IKBKB* in HepG2 cells treated with 10 μM sulforaphane (SFN) for the indicated times (n = 3) **(D)**; or *IKBKG* in HepG2 cells treated with tBHQ and Olt for the indicated times (n = 3) **(E)**. **(F)** ChIP-seq analysis for *IKBKG* antioxidant response element regions in sulforaphane-treated lymphoblastoid cells using the cDNA array database (GSE37589) (upper). *IKBKG* promoter-reporter assays. Luciferase activity was measured in HepG2 cells after Mock or hNrf2 transfection (n = 3 each) (lower left). WT or MT luciferase constructs were used in HepG2 cells after Mock or hNrf2 transfection (n = 3) (lower right). **(G)** NEMO and Nrf2 immunoblottings for SFN- and Olt-treated HepG2 cells for the indicated times (upper). Band intensities represent values relative to the respective control (n = 3 each) (lower). For **B** and** D-G** values were expressed as mean ± SEM (*P < 0.05, **P < 0.01). Statistical significance was tested via two-tailed Student's *t*-tests or one-way ANOVA coupled with Tukey HSD or the LSD multiple comparison procedure when appropriate.

We next extracted 84 genes from Nrf2 KO (GSE8969) and Keap1 KO mice (GSE11287) (Figure 5A, left), and analyzed the microarray datasets for functional GO enrichment to explore the Nrf2 and NEMO association (Figure 5A, middle). DAVID database analysis identified the 'lipid metabolic process' and 'positive regulation of gene expression' biological processes, which shared *Ikbkg* as a core gene in the gene-concept network (Figure 5A, right). In addition, the String program aided in determining *Ikbkg* and *Map4k4* as affected TNFα pathway targets (Figure S6A). As predicted, *Ikbkg* mRNA levels were diminished in Nrf2 KO mice (GSE8969) but were increased in Keap1 KO mice (GSE11287) (Figure 5B). In APAP-induced acute liver failure patients (GSE74000), *IKBKG* levels decreased, and Nrf2 target genes were also repressed (i.e., *HMOX1*, *PRDX1*, *TXNRD1*, *GPX4*, *GPX1*, *GCLC*, *GCLM*, and *NQO1*) (Figure 5C).

Then, we explored the potential transcriptional activation of Nrf2 for the *IKBKG* gene regulation. Sulforaphane (SFN, an Nrf2 inducer)-treated HepG2 cells exhibited increased *IKBKG* mRNA levels but not those of *CHUK* or *IKBKB* (Figure 5D). Other Nrf2 activators (i.e., tert-butylhydroquinone [tBHQ] and oltipraz [Olt]) exerted the same effects (Figure 5E). We also confirmed the nuclear accumulation of Nrf2 by the Nrf2 activators (SFN, Olt, and tBHQ) in HepG2 cells (Figure S6B). Likewise, these compounds increased *Ikbkg* mRNA levels in AML12 cells (Figure S6C).

Analyzing a publicly accessible Nrf2 ChIP-sequencing (ChIP-seq) dataset, two putative antioxidant response elements (AREs) were determined as well-conserved regions across the species. Additionally, SFN treatment induced a strong peak in ARE2 located in the proximal promoter (-112 bp ~ -103 bp) but not in ARE1 (-1003 bp ~ -994 bp) (Figure 5F, upper). Increased H3K27ac and Pol II ChIP intensities validated Nrf2 and ARE2 interactions (Figure S6D). Consistently, enforced Nrf2 expression sharply preponderated *IKBKG* promoter-driven luciferase activity (Figure 5F, lower left). We then crafted specific mutants with mutations at one or both ARE (i.e., ARE1 MT, ARE2 MT, or ARE1/2 MT) for reporter gene assays (Figure 5F, lower right); As expected, hNrf2-inducible luciferase expression was entirely attenuated in ARE2 MT (located between -112 and -103 bp) or ARE1/2 MT reporter assays but not ARE1 MT, verifying ARE2's functional role.

Furthermore, SFN, Olt, and tBHQ treatments begat NEMO protein induction in HepG2 cells (Figure 5G and S6E). Likewise, an Nrf2 deficiency inhibited hepatic NEMO expression (Figure S6F), corroborated by HepG2 cell immunoblottings (Figure S6G). Together, these results firmly evidence that Nrf2 transactivates the *IKBKG* gene.

### The role of the Gα_12_-ROCK1 axis in NEMO regulation

**Figure 6:**
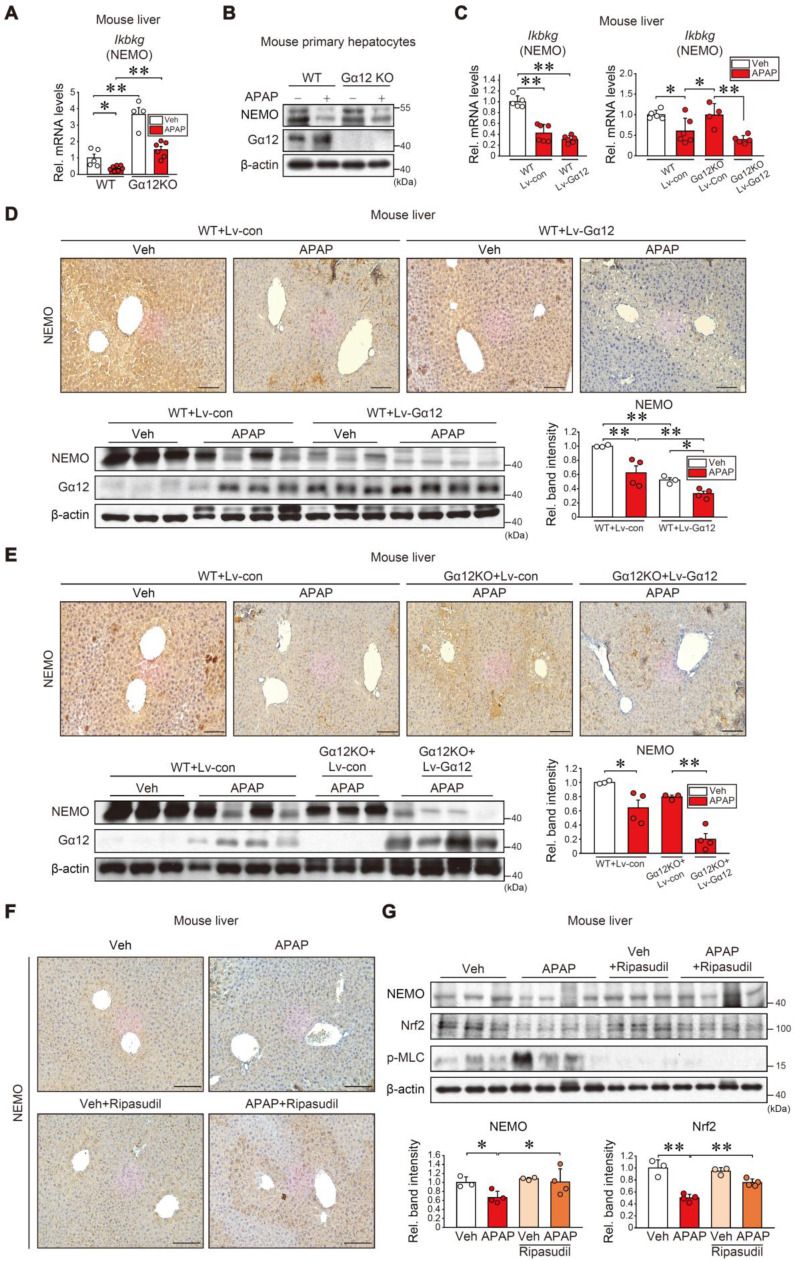
** Gα_12_ modulation effects on NEMO expression. (A)** qRT-PCR assays for *Ikbkg* in WT or Gα_12_ KO mice treated with APAP (300 mg/kg BW, i.p., 6 h) (n = 4-7 each). **(B)** Immunoblottings for NEMO and Gα_12_ in WT or Gα_12_ KO mouse primary hepatocytes treated with APAP (10 mM, 12 h) (n = 3; repeated three times with similar results). **(C)** qRT-PCR assays for *Ikbkg* in WT (n = 5 or 6 each) (left) or Gα_12_ KO mice (n = 4-6 each) (right) treated with APAP (300 mg/kg BW, 6 h) one-week post-injection with Lv-con or Lv-Gα_12_ via tail vein. **(D, E)** Immunohistochemistry (upper) and immunoblottings (lower) for NEMO and Gα_12_ for the same mice as in **C**. Band intensities represent values relative to the respective control (n = 3 or 4 each) (lower right). Scale bar, 200 µm. **(F)** Immunohistochemistry for NEMO in ripasudil-treated WT mice (50 mg/kg BW, 5 h) 1 h after APAP treatment (300 mg/kg BW, 6 h). Scale bar, 200 µm. **(G)** NEMO, Nrf2, and p-MLC immunoblottings (upper) for the same mice as in **F**. Band intensities represent values relative to the respective control (n = 3 or 4 each) (lower). For **A**, **C-E, and G**, values were expressed as mean ± SEM (*P < 0.05, **P < 0.01). Statistical significance was tested via one-way ANOVA coupled with Bonferroni's method or the LSD multiple comparison procedure when appropriate.

Following the observation of significantly inhibited NEMO by APAP-induced ER stress, we further probed into the mechanistic underpinnings of NEMO downregulation induced by APAP, which aimed to elucidate factors beyond NEMO's transcriptional activity. Having previously identified Gα_12_ overexpression by ER stress influences ROCK1-dependent ferroptosis 28, we wondered whether Gα_12_ modulations affect NEMO expression. Notably, Gα_12_ KO enhanced gene sets associated with NF-κB-related pathways in the GSEA analysis regarding our RNA-seq data from WT and Gα_12_ KO mice (GSE173595) (Figure S7A). Also, Gα_12_ deficiency enhanced basal *Ikbkg* mRNA levels in the liver, preventing APAP from inhibiting *Ikbkg* (Figure 6A and Figure S7B). In addition, immunoblotting assays using primary hepatocytes demonstrated that Gα_12_ abrogation considerably reversed APAP's inhibition on NEMO (Figure 6B). Consistently, Gα_12_ overexpression using a liver-specific lentiviral albumin-Gα_12_ (Lv-Alb-Gα_12_) fortified APAP's inhibition on *Ikbkg* mRNA, reversing Gα_12_ KO plus APAP's promotion of the mRNA (Figure 6C). Immunohistochemistry and immunoblottings further confirmed that Gα_12_ overexpression inhibited NEMO in the liver of WT and Gα_12_ KO mice challenged by APAP (Figures 6D and E).

Considering that ROCK1 is a downstream Gα_12_ signaling effector, we further examined whether ROCK1 modulations impacted NEMO levels. ROCK1 knockdown and chemical inhibition (ripasudil) prevented APAP from inhibiting NEMO in primary hepatocytes (Figure S7C). Immunohistochemistry and immunoblottings corroborated ripasudil's influence on NEMO in the liver (Figure 6F and G) while also ameliorating liver injury (Figure S7D). Nrf2 and p-MLC levels were accordingly changed by a downstream ROCK1/2 effector; Ripasudil's ability to heighten NEMO levels was seemingly greater than Nrf2, indicating that the Gα_12_ pathway may additionally regulate NEMO (Figure 6G). Together, these results support that the Gα_12_-ROCK1 axis negatively controls NEMO under liver injury conditions.

### miR-125a inhibition of NEMO downstream from Gα_12_

**Figure 7:**
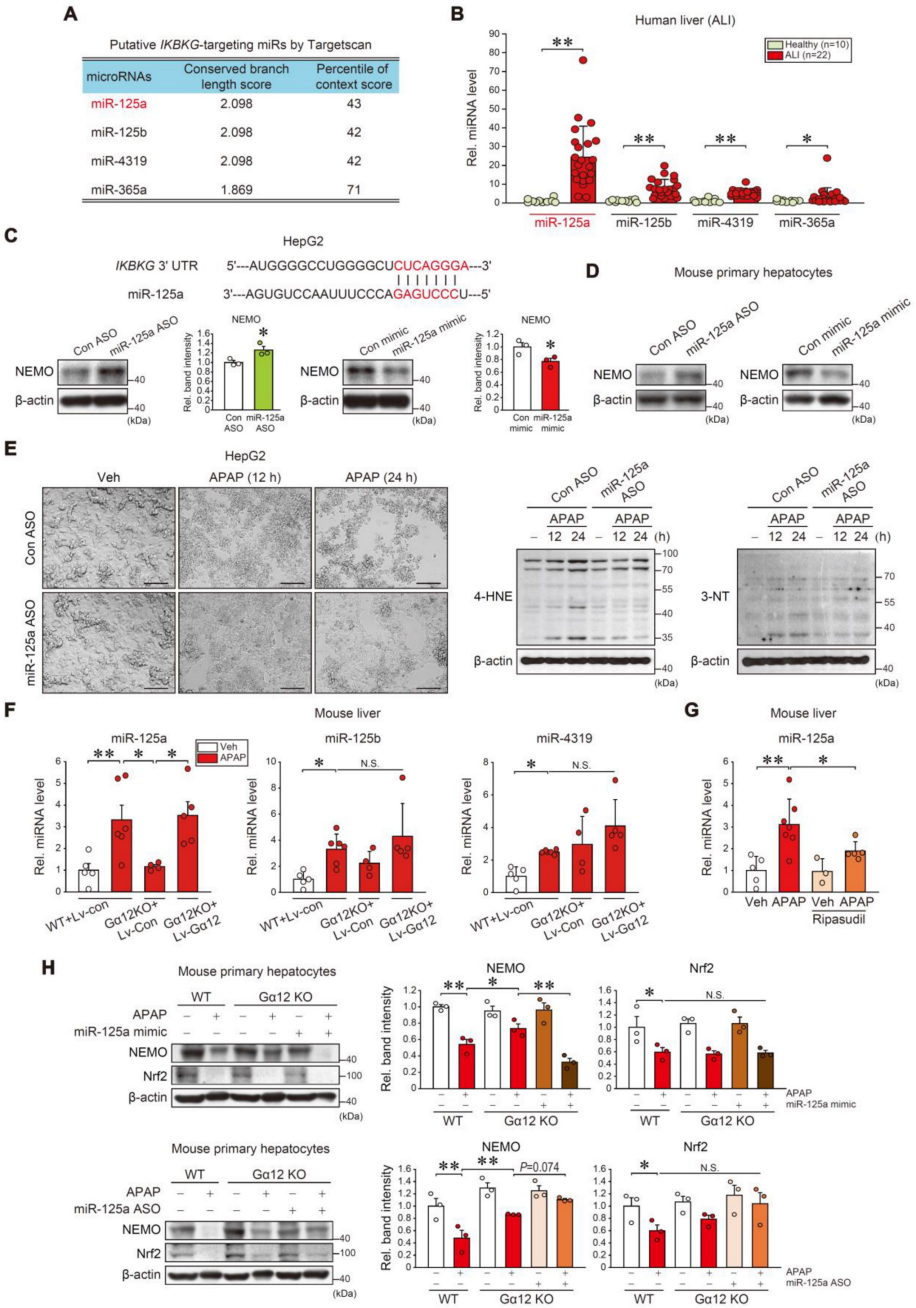
** miR-125a as a downstream NEMO inhibitor regulated by Gα_12_. (A)** Putative *IKBKG-*targeting miRNA candidates were obtained according to conserved branch length and context percentile scores using Targetscan. **(B)** qRT-PCR assays for miR-125a, miR-125b, miR-4319, and miR-365a in healthy individuals (n = 10) or ALI patients (n = 22). **(C)** Prediction of miR-125a binding to the 3'UTR of *IKBKG* mRNA (upper). Immunoblottings for NEMO in HepG2 cells transfected with miR-125a ASO (control ASO) (100 nM, 48 h) (lower left) or miR-125a mimic (control mimic) (100 nM, 48h) (lower right). Densitometric band intensities represent values relative to the respective control (n = 3 each). **(D)** Immunoblottings for NEMO in primary hepatocytes transfected with miR-125a ASO (control ASO) (100 nM, 48 h) (left) or miR-125a mimic (control mimic) (100 nM, 48 h) (right) (n = 3; repeated three times with similar results). **(E)** Representative cell morphology (left) and immunoblottings for 4-HNE and 3-NT (right) in HepG2 cells treated with APAP (20 mM, 12 or 24 h) after miR-125a ASO (or control ASO) transfection (100 nM, 48 h) (n = 3; repeated three times with similar results). Scale bar, 100 µm. **(F)** qRT-PCR assays for miR-125a, miR-125b, and miR-4319 in the same mice as in **Fig. 6E** (n = 4-6 each). **(G)** qRT-PCR assay for miR-125a in ripasudil-treated WT mice livers (50 mg/kg BW, 5 h) 1 h after APAP treatment (300 mg/kg BW, 6 h) (n = 3-7). **(H)** Immunoblottings for NEMO and Nrf2 in WT and Gα_12_ KO mouse primary hepatocyte treated with APAP (10 mM, 12 h) after transfection with miR-125a mimic (upper) or ASO (lower). Densitometric band intensities represent values relative to the respective control (n = 3 each) (right). For **B**, **C**, and **F-H** values were expressed as mean ± SEM (*P < 0.05, **P < 0.01). Statistical significance was tested via two-tailed Student's *t*-tests, Mann-Whitney U test, or one-way ANOVA with the LSD multiple comparison procedure when appropriate.

Next, we aimed to ascertain how microRNA (miRNAs) were involved in post-transcriptional NEMO regulation downstream from the Gα_12_ pathway. Four miRNAs with conserved sites and the potential to bind to *IKBKG* mRNA's 3'-UTR were selected using the 'Targetscan' database (Figure 7A). Out of the four, miR-125a was the most significantly elevated in ALI patient livers (Figure 7B). Then, miR-125a's effect on the *IKBKG* 3'-UTR was examined. *IKBKG* 3'-UTR and the miR-125a seed sequence exhibit a nearly complete pairing (Figure 7C, upper). When assessing miR-125a modulation effects on NEMO protein, miR-125a antisense oligonucleotides (ASOs) increased NEMO levels in HepG2 cells, whereas its mimic exerted the opposite (Figure 7C, lower). This effect was confirmed in primary hepatocytes (Figure 7D). Furthermore, GO analysis was accomplished to distinguish downstream pathways and the functional role of miR-125a-targeted genes. The target genes correlated with the MAPK and NF-κB pathways (Figure S8A, left), which were then implemented in a GO analysis (red color), establishing that they were correlated with the 'Apoptotic process' (the most significantly affected) (Figure S8A, right and S8B). Further, we verified miR-125a's functional role in ferroptosis elicited by APAP or Erastin treatment. As expected, APAP treatment induced morphological changes in HepG2 cells and primary hepatocytes, which were ameliorated by miR-125a ASO pretreatment (Figure 7E, left, S8C, and S8D). APAP's ferroptosis biomarkers (i.e., 4-HNE and 3-NT) were also impeded by miR-125a ASO transfection (Figure 7E, right), confirming miR-125a's effect on ferroptosis processes.

Then, we observed APAP treatment increased miR-125a, miR-125b, and miR-4319 levels in mice livers. Gα_12_ KO completely inhibited APAP's effect on miR-125a but not the others (Figure 7F); This finding is also evidenced by the absence of Gα_12_ regulatory effect on miR-125b in the Gα_12_QL microarray dataset analysis (Figure S9), indicating the specific Gα_12_ signaling effect on miR-125a. In addition, this inhibitory effect was entirely reversed through enforced Gα_12_ expression. Using ripasudil to inhibit ROCK1 resulted in similar outcomes (Figure 7G). In the experiment using WT and Gα_12_ KO primary hepatocytes, a deficiency of Gα_12_ prevented APAP from inhibiting NEMO, which was completely abrogated by miR-125a mimic transfection, but increased by miR-125a ASO (Figure 7H). Intriguingly, Nrf2 levels were not changed. Overall, our results fortify the concept of the dual NEMO regulatory pathways; (1) Nrf2 transcriptionally induces NEMO, and (2) ER stress-mediated Gα_12_ overexpression inhibits NEMO *de novo* synthesis by enhancing miR-125a level, which can be countered by ROCK1 inhibition.

### Dysregulation of NEMO, Nrf2, GPX4 and miR-125a in patients with ALI

**Figure 8:**
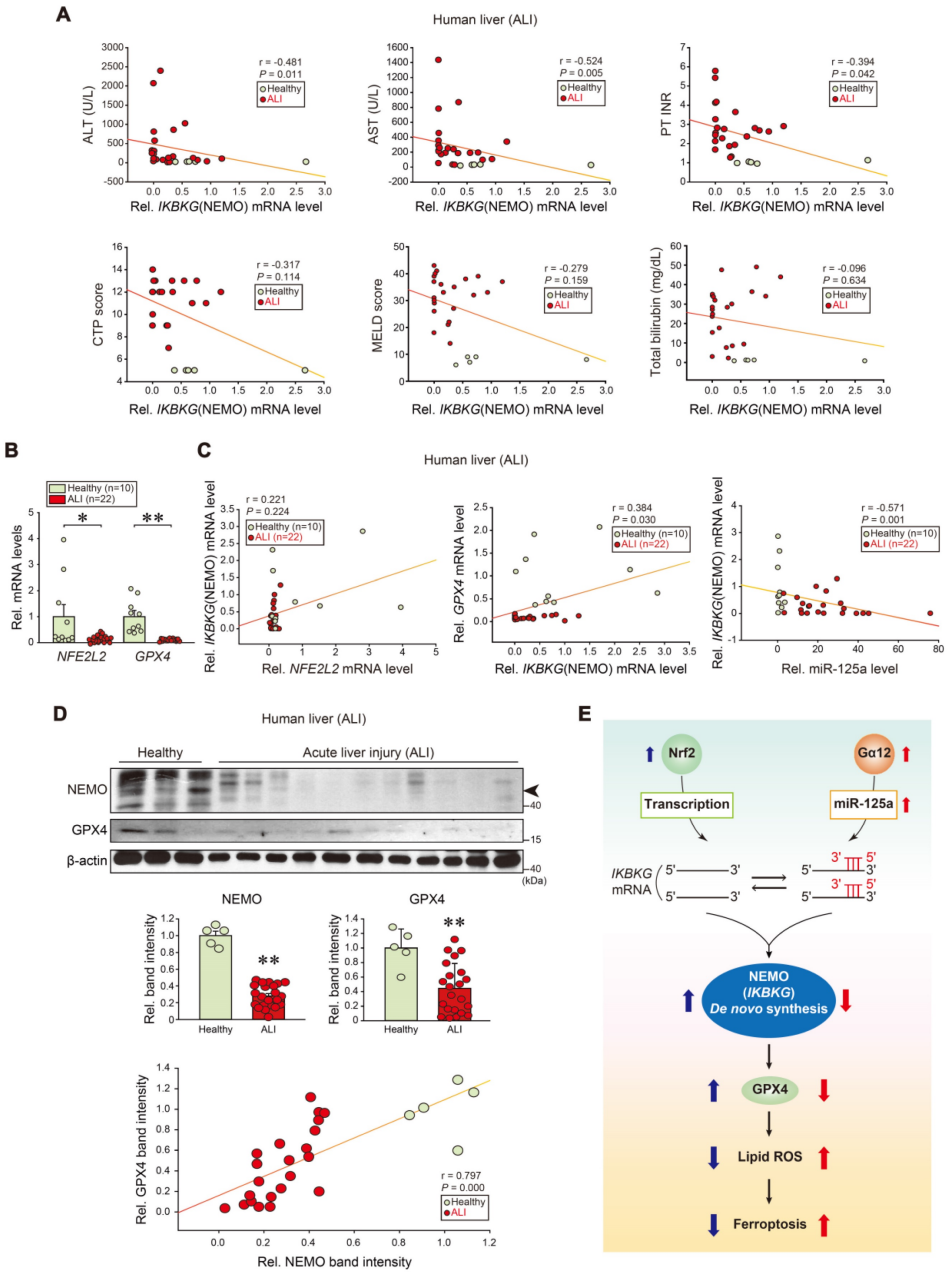
** Correlations between human NEMO and liver disease scores, and identified target levels in patients with ALI. (A)** Correlations between *IKBKG* transcript and ALT, AST, PT INR, CTP, MELD scores, or total bilirubin contents in livers of healthy individuals or ALI patients (healthy: n = 5, ALI: n = 22). ALT, alanine aminotransferase; AST, aspartate aminotransferase; PT INR score, prothrombin time international normalized ratio. CTP, child-turcotte-pugh; MELD score, model for end-stage liver disease score. **(B)** *NFE2L2* and* GPX4* transcript levels in livers of healthy individuals (n = 10) or ALI patients (n = 22). **(C)** Correlations between *NFE2L2* and *IKBKG* (left), or *IKBKG* and *GPX4* (middle), or miR-125a and *IKBKG* transcripts (right) in livers of healthy individuals or ALI patients (healthy, n = 10; ALI, n = 22). **(D)** NEMO and GPX4 immunoblottings for healthy individuals or ALI patients (upper). Band intensities represent values relative to each respective control (healthy: n = 5, ALI: n = 22) (middle). Representative blots were shown. A correlation between NEMO and GPX4 protein levels (n = 27) (lower). **(E)** A schematic depicting how Nrf2-dependent NEMO induction inhibits liver injury through GPX4 against the Gα_12_-miR-125a axis. For **B** and** D**, values were expressed as mean ± SEM (*P < 0.05, **P < 0.01). Statistical significance was tested via two-tailed Mann-Whitney U test or Spearman's correlation analyses.

To further assess the association of *IKBKG* transcript levels and liver injury in clinical situations, we analyzed samples of patients with ALI on drugs. Significant negative correlations existed between *IKBKG* and each ALI marker: alanine aminotransferase (ALT), aspartate aminotransferase (AST), prothrombin time international normalized ratio (PT INR), Child-Turcotte-Pugh score (CTP score), MELD score, and total bilirubin contents (Figure 8A). As expected, *NFE2L2* and *GPX4* transcript levels were decreased in ALI patients (Figure 8B). In patients with ALI or fulminant liver failure, positive correlations existed between *IKBKG* and *NFE2L2*; or *IKBKG* and *GPX4* transcripts. Hepatic miR-125a negatively correlated with *IKBKG* (Figure 8C). We also confirmed the inhibition of NEMO and GPX4 protein levels in patients with ALI, and correlations between NEMO and GPX4 (Figure 8D). In summary, NEMO and GPX4 levels were decreased in the patients through the dual regulatory pathways, exacerbating hepatocyte ferroptosis and ALI (Figure 8E).

## Discussion

The worldwide surge of severe liver injury highlights the necessity for identifying new drug targets and therapeutic compounds to treat this disease 11. Recent reports have indicated that lipid peroxide and oxidative stress accumulations, as observed in ferroptosis, contribute to hepatocyte injury and trigger inflammatory responses, exacerbating liver disease progression 8. Developing medications to regulate ferroptosis has garnered substantial attention 35; however, studies on ferroptosis and its underlying mechanisms are currently insufficient. Hence, future investigations must identify novel regulators and elucidate the underlying mechanisms behind ferroptosis modulation relative to liver injury.

The NF-κB pathway is vital in regulating immune and inflammatory responses in the liver, thereby exerting considerable influence on liver homeostasis and disease processes. The canonical NF-κB activation pathway involves the IKK complex 36, whereas the non-canonical pathway incorporates NF-κB inducing kinase (NIK) upon receptor ligation 37. However, emerging evidence has highlighted that NEMO, a prominent NF-κB activation protein, can protect hepatocytes independent of the canonical NF-κB pathway 38.

Our study uncovered a critical finding expounding upon NEMO's inhibitory role in ferroptosis during APAP intoxication, supporting NF-κB and its influence in further cell fate determination. Our findings showed that 3-NT and 4-HNE intensity changes and their reversal through NEMO overexpression support the ability of NEMO to protect hepatic cells from ferroptosis. Specifically, the outcomes of this study introduce a previously uncharacterized role of NEMO transcriptionally activated by Nrf2 and NEMO induction which inhibits hepatic ferroptosis via GPX4. Our findings indicate that NEMO levels are additionally affected by the Gα_12_ axis responsible for an increase of miR-125a as a previously unknown NEMO inhibitor. These findings establish significant links not only between Nrf2 and NEMO but also between NEMO and miR-125a controlled by the Gα_12_-ROCK1 axis.

We found that NEMO depletion causes upregulation of various gene clusters, including those involved in redox state and arachidonic acid metabolism, showing that NEMO may have a more substantial role in regulating cellular homeostasis beyond its well-known function in NF-κB 39. The reactive intermediate NAPQI, produced from APAP biotransformation, depletes cellular GSH content and binds to cellular macromolecules in hepatocytes 8, effectuating ferroptosis, as confirmed in the present study. Our study also highlights the importance of GPX4 and its cofactor GSH in removing lipid peroxides and oxidation products, such as phospholipids and PUFAs 9, enabling hepatocytes to survive GSH depletion caused by BSO and Erastin treatment 40. Furthermore, this study strongly evidenced that NEMO participates in cell fate determination mediated by GPX4, which alters the glutathione/oxidized glutathione disulfide (GSH/GSSH) ratio. This notion is corroborated by DCF-DA intensity alterations upon APAP exposure following NEMO overexpression.

The PERK/CHOP-dependent JNK pathway has been proven to induce cell death in response to ER stress 41, 42. Our findings demonstrate that NEMO overexpression inhibits p-PERK and CHOP upregulation mediated by ER stress, suggesting NEMO's potential in attenuating ER stress-induced hepatocyte injury. However, ALT activity and TUNEL staining intensity changes after NEMO overexpression were more considerable than those in p-PERK and CHOP, indicating that other ER stress mediators may also be involved. IRE1α, a known Gα_12_ overexpression 28 and eIF2α regulator through PERK 43, potentially contributes to p-PERK/CHOP regulation under APAP intoxication conditions, even when NEMO is present. Therefore, NEMO likely regulates ferroptosis by inhibiting the PERK/CHOP-dependent JNK pathway and interacting with other mediators to regulate overall cellular responses to ER stress.

APAP-induced liver injury activates the apoptosis pathway and various other regulated necrosis types, namely necroptosis, and pyroptosis 44-46. Among diverse and complex cell death pathways, JNK is an integral stress kinase in APAP-induced liver injury and hepatocyte death as it activates RIP1 and engenders necrosome complex formation 47, 48. Furthermore, necroptosis is a programmed necrosis triggered by RIP1, RIP3, and MLKL. Previous studies have demonstrated that NEMO overexpression attenuates p-JNK and RIPK1 activation, preventing the RIPK1/FADD/caspase-8 complex from forming to inhibit necrotic cell death 18, consistent with our findings that NEMO overexpression prevents APAP from increasing p-JNK, p-RIP1, p-RIP3, and p-MLKL. In the present study, other gene clusters affected by NEMO depletion include inflammatory response pathways, being consistent with our previous observation confirming JNK's role in NF-κB-mediated inflammatory responses 33.

Our study identified Nrf2 as an *IKBKG* gene transcription factor, revealing a previously unknown NF-κB pathway regulation. Additionally, the observation that Nrf2 activation upregulates Bcl-2 and other Nrf2-target genes confirmed that Nrf2 could protect cells from chemical stresses while maintaining cellular homeostasis 24. Nrf2 is not only imperative for cell survival as a nuclear receptor but also as a direct regulator of target genes, holding significant scientific importance concerning liver injuries. Moreover, Nrf2 can modulate key cytoprotective and antioxidant enzyme expressions pivotal in mitigating liver damage and promoting tissue repair 49. Understanding the direct Nrf2 regulation of target genes provides insights into molecular mechanisms underlying liver injury and offers potential targets for developing interventions to combat liver pathologies.

Notably, the previous study demonstrated that Gα_12_ overexpression was associated with ER stress in acute liver injury through ROCK1, mediated by ALOX12 and miR-15a dysregulation 28. This study also aimed to elucidate the newly acknowledged targets downstream from the Gα_12_ axis. Our results isolate prominent mechanisms underlying the Nrf2-dependent NEMO-GPX4 pathway's protective effect against ER stress, aligning with the previous report on Nrf2 activation through Gα_12_ deficiency 50. Interestingly, ripasudil treatment prompted a substantial NEMO elevation against APAP intoxication but only weakly increased Nrf2 levels. This observation indicates that the Gα_12_-ROCK1 axis plays a distinct NEMO regulatory role, identified by ROCK1-mediated translational control separate from Nrf2-dependent NEMO induction.

Additional efforts enabled us to identify miR-125a as a functional NEMO inhibitor downstream from Gα_12_. Moreover, our data demonstrating miR-125a inhibition of *IKBKG* mRNA translation into proteins correlates with ROCK1-mediated inhibitory protein regulation. The different NEMO and Nrf2 levels after ripasudil treatment corroborate miR-125a involvement in NEMO expression downstream from Gα_12_. Also, apoptosis with JNK activation initiates from miR-125a targeted gene clusters associated with the NF-κB pathway, with *IKBKG* as the core gene. When analyzing miR-125a target genes using GO, we extracted the Biocarta pathway of the 'MAP kinase signaling pathway' and 'NF-κB signaling pathway' (red color) and determined *IKBKG* as a core gene clustering with the 'JNK phosphorylation and activation mediated by activated human TAK1' pathway (Figure S8B). Thus, the miR-125a-associated gene network may link to the JNK pathway for NEMO regulation.

Gaining a comprehensive understanding of non-canonical NEMO regulation pathways would be crucial to insights into therapeutic interventions. This study's RNA-seq analyses, human sample analyses, *in vivo,* and *vitro*-based outcomes established that ALI downregulated NF-κB and its related pathways. In addition, our findings confirmed that NEMO expression was diminished in ALI patients and was negatively correlated with representative liver injury markers. Consistently, we observed that miR-125a levels were significantly increased in the patient samples, matching miR-125a upregulation in the livers of mice with fibrosis 51. Moreover, the close associations between *NFE2L2* and *IKBKG*, or *IKBKG* and *GPX4*, or miR-125a and *IKBKG* in our ALI sample analyses further support these pathways' prominence in human liver health and diseases.

Collectively, our research reveals that NEMO mitigates hepatocytes ferroptosis by inducing GPX4. This effect is achieved through the inhibition of both chemical-induced ROS production and ER stress. Furthermore, the outcomes have illuminated the role of Nrf2 as a transcriptional regulator of NEMO. We have also uncovered miR-125a as an unprecedented suppressor of NEMO *de novo* synthesis, acting downstream from the Gα_12_-ROCK1 axis. Together, our data evidence the dual antagonistic NEMO regulation by Nrf2-dependent transcription and ROCK1-mediated microRNA regulation of protein synthesis (Figure 8E). Thus, the identified molecules will likely work together for ER stress-mediated ferroptosis. These newly identified pathways and targets present implications for developing therapeutic strategies aiming at ALI.

## Supplementary Material

Supplementary materials and methods, figures and table.

## Abbreviations

3-NT: 3-nitrotyrosine
4-HNE: 4-hydroxynonenal
ALF: acute liver failure
ALI: acute liver injury
ALT: alanine aminotransferase
APAP: acetaminophen
AREs: antioxidant response elements
ASK1: apoptosis signal-regulating kinase 1
ASO: antisense oligonucleotide
AST: aspartate aminotransferase
ATF6: activating transcription factor 6
BSO: buthionine sulfoximine
CCl4: carbon tetrachloride
ChIP-seq: chromatin immunoprecipitation
CHOP: C/EBP-homologous protein
CTP score: Child-Turcotte-Pugh score
CYP2E1: cytochrome P450 2E1
DCF-DA: 2′,7′-dichlorofluorescein diacetate
DEGs: differentially expressed genes
ER: endoplasmic reticulum
FADD: fas-associated death domain protein
FDR: false discovery rate
Fer-1: ferrostatin-1
Gα_12_: G protein subunit alpha 12
GEO: Gene Expression Omnibus
GO: Gene Ontology
GPX4: glutathione peroxidase 4
GRP78: glucose regulatory protein 78
GSH: glutathione
GSEA: gene set enrichment analysis
GSSG: oxidized glutathione disulfide
HBV-ALF: HBV-associated acute liver failure
IKK: IκB kinase
IRE1α: inositol-requiring enzyme 1α
JNK: c-Jun NH2-terminal kinase
KEGG: Kyoto Encyclopedia of Genes and Genomes
Lv-Alb- Gα_12_: lentiviral albumin-Gα_12_
MAPK: mitogen-activated protein kinase
MELD score: model for end-stage liver disease score
MHN: massive hepatic necrosis
miR-125a: microRNA-125a
miRNA: microRNA
MKK4: mitogen-activated protein kinase kinase 4
MLKL: mixed lineage kinase domain like pseudokinase
NAPQI: N-acetyl-p-benzoquinone imine
NES: normalized enrichment score
NEMO: NF-κB essential modulator
NF-κB: nuclear factor kappa-light-chain-enhancer of activated B cells
NIK: NF-κB inducing kinase
Nrf2: Nuclear factor erythroid-2-related factor2
Olt: oltipraz
PAMPs: pathogen-associated molecular patterns
PCA: principal component analysis
PERK: protein kinase R-like endoplasmic reticulum kinase
p-MLC: phosphor-myosin light chain
PT INR: prothrombin time international normalized ratio
PUFAs: polyunsaturated fatty acids
RIPK1: receptor-interacting protein kinase 1
RIPK3: receptor-interacting serine/threonine-protein kinase 3
RNA-seq: RNA sequencing
ROCK1: rho associated coiled-coil containing protein kinase 1
ROS: reactive oxygen species
SFN: sulforaphane
SHN: submassive hepatic necrosis
siRNA: small interfering RNA
TAK1: transforming growth factor-β-activated kinase 1
tBHQ: tert-butylhydroquinone
Tm: tunicamycin
TNFα: tumor necrosis factor-α
TUNEL: terminal deoxynucleotidyl transferase dUTP nick end labeling
UPR: unfolded protein response
UTR: untranslated region
WT: wild type
