# Supplementary material for: Dual regulation of NEMO by Nrf2 and miR-125a inhibits ferroptosis and protects liver from endoplasmic reticulum stress-induced injury
Source: Theranostics. 2024 Feb 24;14(5):1841–59. doi: 10.7150/thno.89703 (PMC10945339; doi:10.7150/thno.89703)
Supplement: Supplementary file 1 — Supplementary materials and methods, figures and table. [file thnov14p1841s1.pdf]

Supplementary Information

Dual regulation of NEMO by Nrf2 and miR-125a inhibits ferroptosis and protects liver from endoplasmic reticulum stress-induced injury

Jihoon Tak, Min Sung Joo, Yun Seok Kim, Hyun Woo Park, Chang Hoon Lee, Gil-Chun Park, Shin Hwang*, and Sang Geon Kim*

Table of contents

Supplementary materials and methods…………………………………………………………....**2**

Supplementary references……………………………………………………………………………**10**

Supplementary figures……………………………………………………………………….......**11**

Supplementary table……………………………………………………………………………..**26**

Supplementary materials and methods

Materials

Antibodies directed against IKKγ (NEMO, sc-8032, sc-166398), IRE1α (sc-390960), GPX4 (sc-166570), and GADD 153 (CHOP, sc-7351) were purchased from Santa Cruz Biotechnology (Santa Cruz, CA). Anti-Nrf2 (ab62352), anti-GRP78 (ab21685), anti-4-hydroxynonenal (ab46545), and anti-3-nitrotyrosine (ab61392) antibodies were supplied from Abcam (Cambridge, UK), whereas anti-Nrf2 (D1Z9C, 12721), anti-phospho PERK (Thr980, 3179), anti-PERK (3192), anti-ATF6 (65880), anti-phospho-JNK (Thr183/Tyr185, 9251), anti-JNK (9252), anti-RIP (3493), anti-phospho-RIP (Ser166, 31122), anti-RIP3 (D4G2A, 95702), anti-phospho-RIP3 (Thr231/Ser232, 91702), anti-phospho-MLKL (37333), anti-ROCK1 (C8F7, 4035), and anti-phospho MLC (Ser358, 91689) antibodies were from Cell Signaling Technology (Danvers, MA). Anti-phospho PERK (Thr980, BS-3330R) was obtained from Bioss (Massachusetts, USA). Horseradish peroxidase-conjugated goat anti-rabbit (G-21234), and goat anti-mouse (G-21040) IgGs were from Invitrogen (Carlsbad, CA). Ripasudil (K-115) hydrochloride dehydrate (S7995) was obtained from Selleckchem (Houston, TX), whereas oltipraz was supplied from CJ Corporation. Anti-β-actin antibody (A5441), sulforaphane (S6317), and other reagents were purchased from Sigma-Aldrich (St. Louis, MO).

Bioinformatic analysis

Publicly accessible patients or mouse gene expression data were downloaded from Gene Expression Omnibus (GEO, https://www.ncbi.nlm.nih.gov/geo/; GSE99878, GSE74000, GSE38941, GSE104302, GSE75277, GSE8969, GSE11287, GSE61100, GSE173595, and GSE44079). Differentially expressed genes (DEGs) were then identified using an independent t-test: DEGs were selected as the genes with P-values < 0.01 or 0.05 with absolute fold-change of > 1.5 or 2. The criterion for statistical significance was set at FDR < 0.25. Statistically enriched signaling pathways of clustered DEGs were ranked and categorized according to the 'Gene ontology pathway', 'KEGG pathway', 'Reactome pathway', and 'Wikipathway' using DAVID 6.8 software, DAVID Knowledgebase v2022q2,

and DAVID Knowledgebase v2022q4 (https://david.ncifcrf.gov/). Each gene represented by an individual dot in a principal component analysis (PCA), volcano plot, and a heat map of significantly expressed genes were obtained by GraphPad Prism 9.5.0 and R software/Bioconductor package using ggplot and gplots function for the visualization.

For the pathway enrichment analyses, the top 10 GO terms based on FDR-corrected *P*-value were represented by bubble plot analysis, whereas the Reactome pathway and associated genes were shown using the Circos diagram [1]. A Circos plot was used for visualizing the data and information in a circular layout, which was used to show the relationships between significantly enriched pathways and regulated genes with genomic intervals or variations. The interaction between selected gene-encoded proteins was analyzed using the STRING (version: 9.1 and 11.5). Cytoscape (3.4.0 and 3.9.1) software application (http://www.cytoscape.org/) visualized gene interaction networks overlapped with gene expression changes.

Gene set enrichment analysis

Transcriptome data from primary human hepatocytes and mice downloaded from GEO (GSE99878, GSE173595) were analyzed using gene set enrichment analysis (GSEA) 3.0 and 4.3.2 software. 'Biological process of gene ontology (GO)', 'WikiPathways", and 'Reactome pathways' from Molecular Signature Database (MSigDB v6.2 and v2022.1.Hs, http://software.broadinstitute.org/gsea/msigdb) and GSEA leading-edge analysis was employed using GSEA 4.3.2 software with the "Signal2Noise" metric to generate a ranked list and a "gene set" permutation type. FDR was used for the statistical significance assessment of the normalized enrichment score (NES). Gene sets with FDR < 0.25 were considered statistically significant. Heatmap represents the respective leading-edge subsets of the most upregulated genes.

Target gene delivery

The mouse albumin enhancer/promoter construct, kindly provided by Dr. Richard D. Palmiter

(University of Washington), was used to make a lentivirus encoding Gα_{12}; the original elongation factor-1 promoter of pCDH-EF1-multiple cloning site-copepod super green fluorescent protein (copGFP) plasmid (System Biosciences) was replaced with the albumin enhancer/promoter. The coding region of pcDNA3-Gα_{12} was extracted and cloned downstream of the albumin enhancer/promoter, as described previously [2]. The constructs were sequenced to assess the integrity of the insert. For *in vivo* experiments, 100 μl of 1.5×10^7 TU was administered to 10-week-old WT or *Gna12* KO mice through the tail vein. At 7 days after injection, the mice were fasted overnight prior to a single dose of APAP treatment, and tissue samples were acquired 6 h later.

Cell lines and primary hepatocytes

AML12 (a mouse hepatocyte-derived cell line) and HepG2 (a human hepatocyte-derived cell line) cells were purchased from American Type Culture Collection (ATCC) (Rockville, Maryland). AML12 cells were cultured in the DMEM/F-12 medium containing 10% FBS, insulin-transferrin-selenium X (ITSX), dexamethasone (40 ng/ml; Sigma), and antibiotics. The cells with less than 20 passage numbers were used. HepG2 cells were maintained in the DMEM containing 10% FBS, 50 units/ml penicillin, and 50 μg/ml streptomycin. Primary hepatocytes were isolated from C57BL/6 mice under the guidelines of the institutional animal use and care committee [3], and plated in a 6-well dish at a density of 2×10^5 cells/well, and wells with 70% to 80% confluence were used. Briefly, under anesthesia with Zoletil, livers were perfused with Ca^{2+}-free Hank's buffered salt solution (Invitrogen, Carlsbad, CA) for 10 min, followed by continuous perfusion with a 0.1% w/v collagenase (Sigma, Type I). The whole liver was removed, and minced in the phosphate-buffered saline. Mouse hepatocytes were filtered through a 0.2 μm cell strainer (BD Biosciences) and centrifuged at 50 *g* for 2 min (three times). Hepatocytes were harvested into collagen-coated plates in isolation media (Dulbecco's modified Eagle's medium [DMEM], high glucose, supplemented with 10% fetal bovine serum [FBS], 100 units/mL penicillin and 100 μg/mL streptomycin, 15 mM 4-(2-hydroxyethyl) piperazine-1-ethanesulfonic acid [HEPES], and 10 nM dexamethasone). After 3 to 4 h, unattached cells were removed by changing isolation media with

culture media (DMEM, low glucose, supplemented with 10% FBS, 100 units/mL penicillin, and 100 μg/mL streptomycin, 5 mM HEPES, and 10 nM dexamethasone). The attached hepatocytes were used for further analysis. We also treated cell lines derived from hepatocytes with 10 μM sulforaphane (SFN), 30 μM oltipraz (Olt), and 30 μM tert-butylhydroquinone (tBHQ) for the specified durations.

Assays for reactive oxygen species (ROS)

Briefly, HepG2 cells were treated with 20 mM APAP for 12 h, washed with PBS, stained with 20 μM of DCFH-DA solution (ab113851, Abcam) for 45 min at 37 °C in the dark, and were rinsed with the dilution buffer according to the manufacturer's protocol. For the positive control, the cells were treated with 100 μM H_2O_2 for 10 min before staining. The fluorescence intensity was measured at 485/528 nm excitation/emission wavelengths using an FP-8200 spectrofluorometer (Jasco, Easton, MD, USA). Cellular fluorescence was measured using the Image J program (NIH, Bethesda, MD, USA).

RNA isolation and quantitative RT-PCR assays

Total RNA was extracted using Trizol (Invitrogen, Carlsbad, CA) and was reverse-transcribed. The resulting cDNA was amplified by qRT-PCR, as previously described [4]. β-Actin, 18S rRNA, or GAPDH was used as a normalization control. The primer sequences used for qRT-PCR assays are listed in Supplementary Table 1.

Plasmid and small interfering RNA transfection

The plasmids encoding for NEMO and PERK were supplied from Addgene (Cambridge, MA). The empty plasmid, pcDNA3.1, was used for mock transfection. Cells were seeded in 6-well plates and were incubated with the indicated plasmid (1 μg) for transfection using Lipofectamine 2000 (Invitrogen, Carlsbad, CA) according to the manufacturer's instructions. Scrambled control siRNA or siRNA directed against NEMO, Nrf2, PERK, and ROCK1 were supplied from Dharmacon (Lafayette, CO,

USA). The cells were transfected with each small-interfering RNA (siRNA) using Lipofectamine 2000 transfection reagent (Invitrogen, Carlsbad, CA) for 24 h.

Hydrodynamic injection of plasmid in mice

For the *in vivo* experiments, a plasmid encoding for NEMO and control plasmid were prepared using the Endofree-plasmid mega kit (Qiagen, Hilden, Germany). After 1 week of acclimation, 8-week-old C57BL/6 mice were hydrodynamically injected with NEMO or control plasmid (25 μg each) via the tail vein. A total volume equivalent to 10% of the mouse's body weight in PBS was delivered within 5-7 seconds. Three days after the hydrodynamic injection, the mice were fasted overnight prior to a single dose of APAP treatment (300 mg/kg BW, i.p.), and the liver and blood samples were obtained 6 h afterward.

Measurement of glutathione and Fe^{2+} contents

The total GSH and glutathione disulfide (oxidized glutathione, GSSG) in liver homogenates were measured using the GSH/GSSG Ratio Detection Assay Kit (Abcam, ab138881) according to the manufacturer's instruction. The amount of reduced GSH (shown as GSH) was calculated from the total GSH and GSSG. The Fe^{2+} levels in the serum samples were detected using the iron colorimetric assay kit (Abcam, ab83366) according to the manufacturer's protocol.

Luciferase reporter assays

For reporter gene assays, the region containing -2,000 base pairs (bp) of *IKBKG* was cloned into the pGL3 luciferase vector (pGL3-IKBKG). A mutation of Nrf2-ARE1/2 was introduced by replacing the sequence of Nrf2-binding element located between -1003 bp and -994 bp (Mut1); or between -112 bp and -103 bp (Mut2), respectively. The cells were transfected with either pGL3-IKBKG luciferase reporter or pGL3-IKBKG-Nrf2-ARE mutant reporter for 24 h in the presence of Lipofectamine 2000 transfection reagent (Invitrogen, Carlsbad, CA), and luciferase activity was assessed by adding

luciferase assay reagent (Promega, Madison, WI).

Chromatin immunoprecipitation (ChIP) assay

HepG2 cells were treated with sulforaphane for 24 h, and formaldehyde was then added to the cells to a final concentration of 1% for cross-linking of chromatin. The ChIP assay was performed according to the EZ-ChIP assay kit protocol (Upstate Biotechnology, Lake Placid, NY, USA). qRT-PCR was done using the primers flanking the indicated anti-oxidant response elements (AREs) binding sites located in the promoter region of the *IKBKG* gene (ARE1: sense, 5’-ATCGGGACAGACTCCAGAAA-3’ and antisense, 5’-CCTTACCCCACCCCTACTGT-3’; ARE2: sense, 5’-GGGGGCTGCTGAGTTAGTTC-3’ and antisense, 5’-ACATTTCTGGGCTGACTTCC-3’). IgG immunoprecipitation represents negative control. One-tenth of cross-linked lysates served as the input control.

Immunoblot analysis

Cells were centrifuged at 3,000 *g* for 3 min and allowed to swell after the addition of lysis buffer in ice for 1 h. The lysates were centrifuged at 10,000 *g* for 10 min to obtain supernatants. A nuclear extraction kit (#ab113474, Abcam) was used to prepare nuclear extracts according to the manufacturer’s instructions. Proteins were separated by 6%, 7.5%, or 12% sodium dodecyl sulfate-polyacrylamide gel electrophoresis and were transferred onto nitrocellulose membranes (Millipore, Bedford, MA). The membrane was blocked with 5% non-fat dried milk in Tris-buffered saline and Tween 20 (TBST) (20 mM Tris-HCl, 150 mM NaCl, and 0.1% Tween 20, pH 7.5) for 1 h, and incubated overnight with primary antibodies at 4ºC. After washing with TBST buffer, membranes were incubated with a horseradish peroxidase-conjugated anti-mouse IgG secondary antibody for 1 h at room temperature. Bands were visualized using the ECL chemiluminescence system (GE Healthcare, Buckinghamshire, UK). Equal loading of proteins was verified by immunoblotting for β-actin. Quantifications were done by scanning densitometry of the immunoblots and β-actin normalization.

Immunohistochemistry

Mouse liver specimens were fixed in 10% formalin, embedded in paraffin, cut into sections, and mounted on slides. Tissue sections were immunostained with the antibody directed against NEMO. Briefly, the paraffin-embedded sections were deparaffinized with xylene and rehydrated with alcohol series. After antigen retrieval was performed, the endogenous peroxidase activity was quenched. The sections were pretreated with 10% normal donkey serum for 40 min to block nonspecific antibody binding and were incubated with the antibodies of interest overnight at 4°C. The sections were then treated with 2% normal donkey serum for 15 min and incubated with biotin-SP-conjugated affinity pure donkey anti-rabbit IgG for 2 h. The labeling was done by using 3,3'-diaminobenzidine. After mounting with Permount solution, the sections were examined using a light microscope (DMRE, Leica Microsystems, Wetzlar, Germany), and images were acquired with Fluoview-II (Soft Imaging System GmbH, Muenster, Germany) attached to the microscope.

Transfection of miRNA mimic or antisense oligonucleotide

Synthetic miRNA duplexes were synthesized and transfected, as previously described [5]. Briefly, the cells in each well (6-well plates) were transiently transfected with 100 nM of miR-125a mimic or control mimic (GenePharma, China), or 2'-O-methyl miR-125a antisense oligonucleotide (ASO) or respective control ASO using Lipofectamine 2000 transfection reagent (Invitrogen, Carlsbad, CA) for 48 h.

Blood biochemical analysis and histopathology

Serum alanine aminotransferase (ALT) and aspartate aminotransferase (AST) activities were analyzed using Spectrum (Abbott Laboratories, Abbott Park, IL). Hematoxylin and eosin (H&E) staining was done as described [6]. Terminal deoxynucleotidyl transferase-mediated deoxyuridine triphosphate nick-end labeling (TUNEL) assays were carried out using the *in situ* S7100 ApopTAG

apoptosis detection kit from Millipore (Temecula, CA) and the DeadEnd Colorimetric TUNEL System from Promega (Madison, WI) according to the manufacturer's instructions.

References

1. Krzywinski M, Schein J, Birol I, Connors J, Gascoyne R, Horsman D, et al. Circos: an information aesthetic for comparative genomics. Genome Res. 2009; 19: 1639-45.

2. Kim TH, Yang YM, Han CY, Koo JH, Oh H, Kim SS, et al. Gα12 ablation exacerbates liver steatosis and obesity by suppressing USP22/SIRT1-regulated mitochondrial respiration. J Clin Invest. 2018; 128: 5587-602.

3. Yang YM, Seo SY, Kim TH, Kim SG. Decrease of microRNA-122 causes hepatic insulin resistance by inducing protein tyrosine phosphatase 1B, which is reversed by licorice flavonoid. Hepatology. 2012; 56: 2209-20.

4. Yang YM, Lee WH, Lee CG, An J, Kim ES, Kim SH, et al. Gα12 gep oncogene deregulation of p53-responsive microRNAs promotes epithelial-mesenchymal transition of hepatocellular carcinoma. Oncogene. 2015; 34: 2910-21.

5. Zhang Q, Pu R, Du Y, Han Y, Su T, Wang H, et al. Non-coding RNAs in hepatitis B or C-associated hepatocellular carcinoma: potential diagnostic and prognostic markers and therapeutic targets. Cancer Lett. 2012; 321: 1-12.

6. Han CY, Lim SW, Koo JH, Kim W, Kim SG. PHLDA3 overexpression in hepatocytes by endoplasmic reticulum stress via IRE1-Xbp1s pathway expedites liver injury. Gut. 2016; 65: 1377-88.

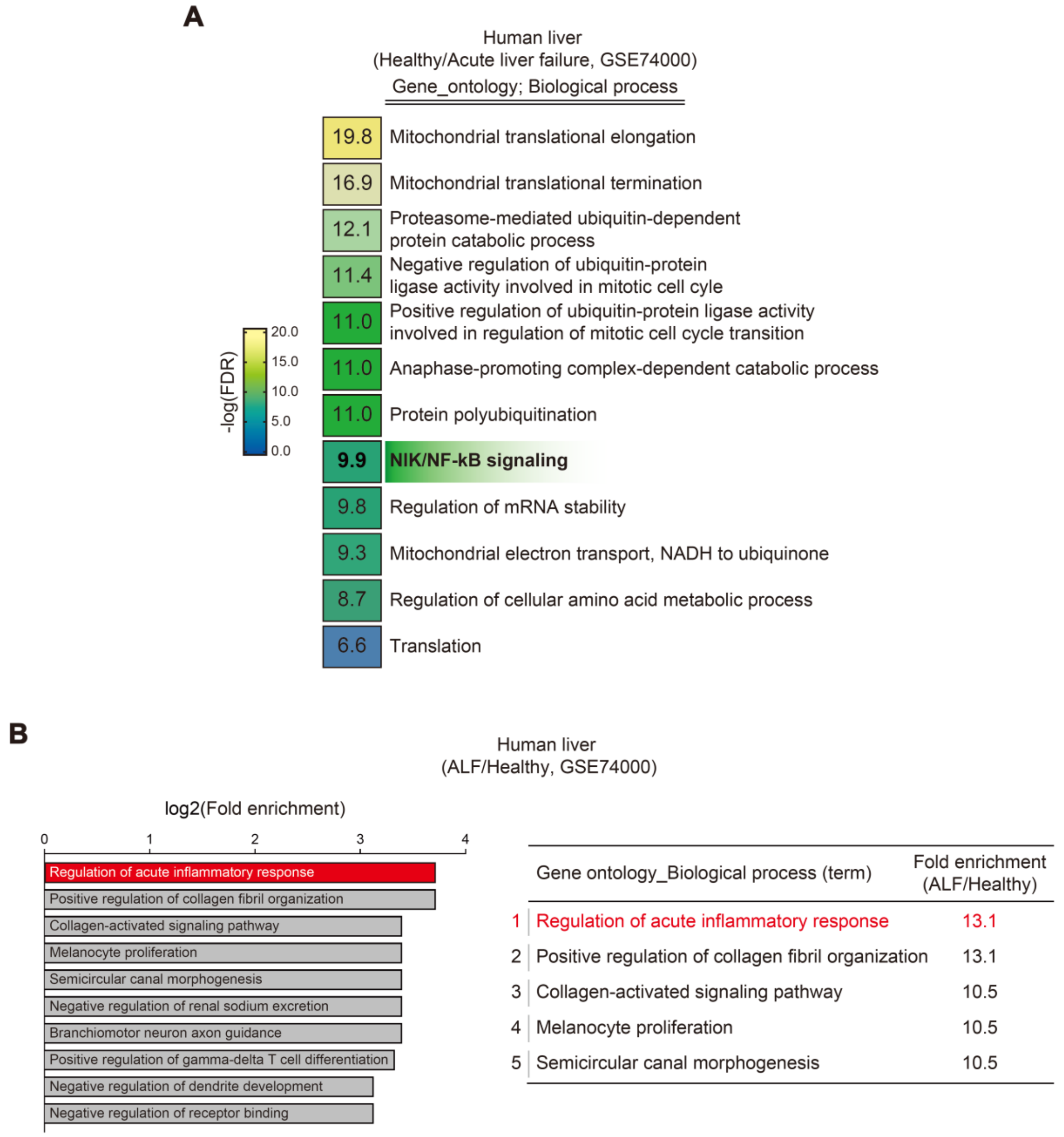

Figure S1. Downregulated NF-κB signaling with an inflammatory response in the livers of patients with acute liver failure

(A) Gene ontology (GO) analysis using microarray dataset in patients with acute liver failure (GSE74000) and the major biological processes using the DAVID bioinformatics database. NF-κB signaling process was negatively changed in patients with acute liver failure (vs. healthy individuals, FC < 0.5 and raw $P < 0.05$).

(B) GO term associated with the regulation of acute inflammatory response in patients with acute liver failure (vs. healthy individuals, fold change > 1.5 and raw $P < 0.05$).

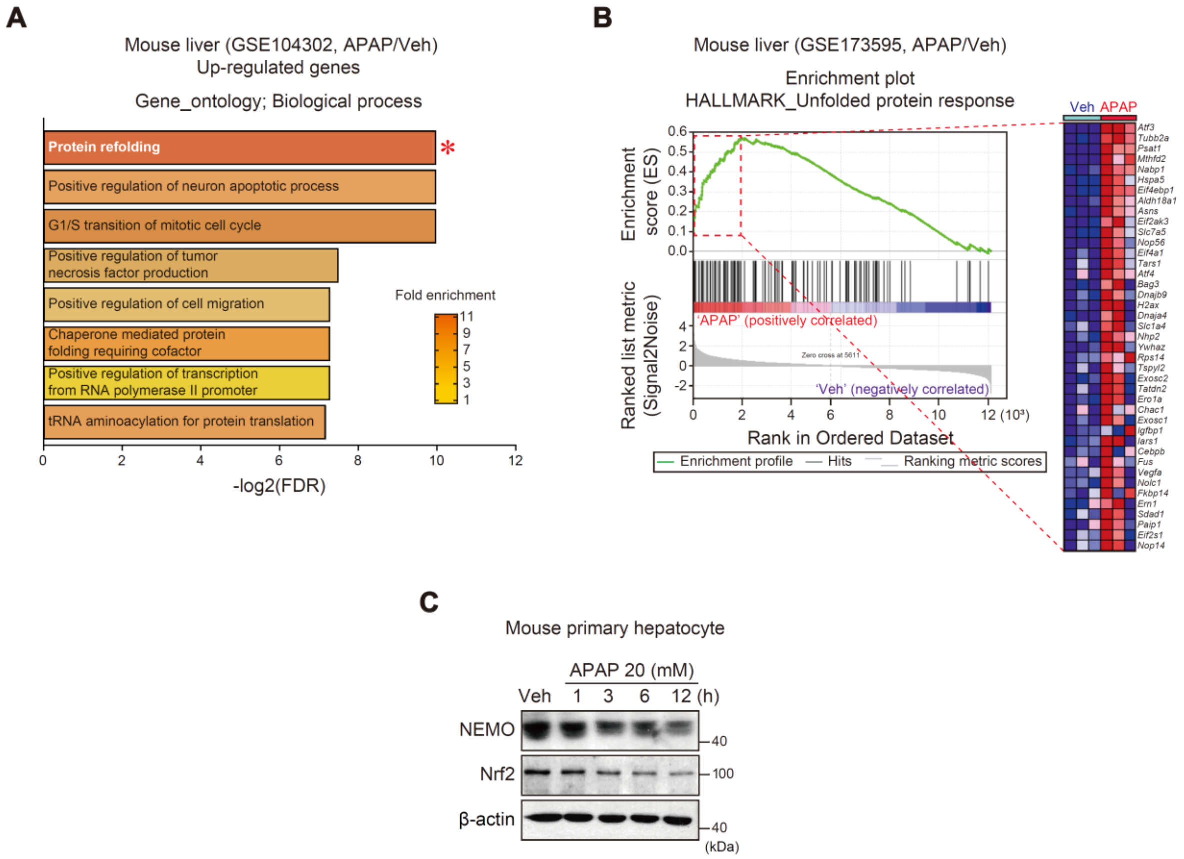

Figure S2. The inhibitory effects of APAP on NEMO and Nrf2 in association with the unfolded protein response

(A) Gene ontology analysis of major top 8 biological processes in the liver of mice treated with APAP (300 mg/kg BW, i.p., 6 h). The white color word in the bar graph of the first rank (red asterisk) indicates the GO term relevant to ER stress (GSE104302, n = 3 each). FDR is shown in the bar graph.

(B) GSEA enrichment plot of the Hallmark category for the unfolded protein response (left) and the leading-edge genes (right) (red-dotted) in mice treated with APAP (n = 3 each) (NES = 2.17, FDR < 0.0001).

(C) Immunoblottings for NEMO and Nrf2 in mouse primary hepatocytes treated with 20 mM APAP for the indicated times.

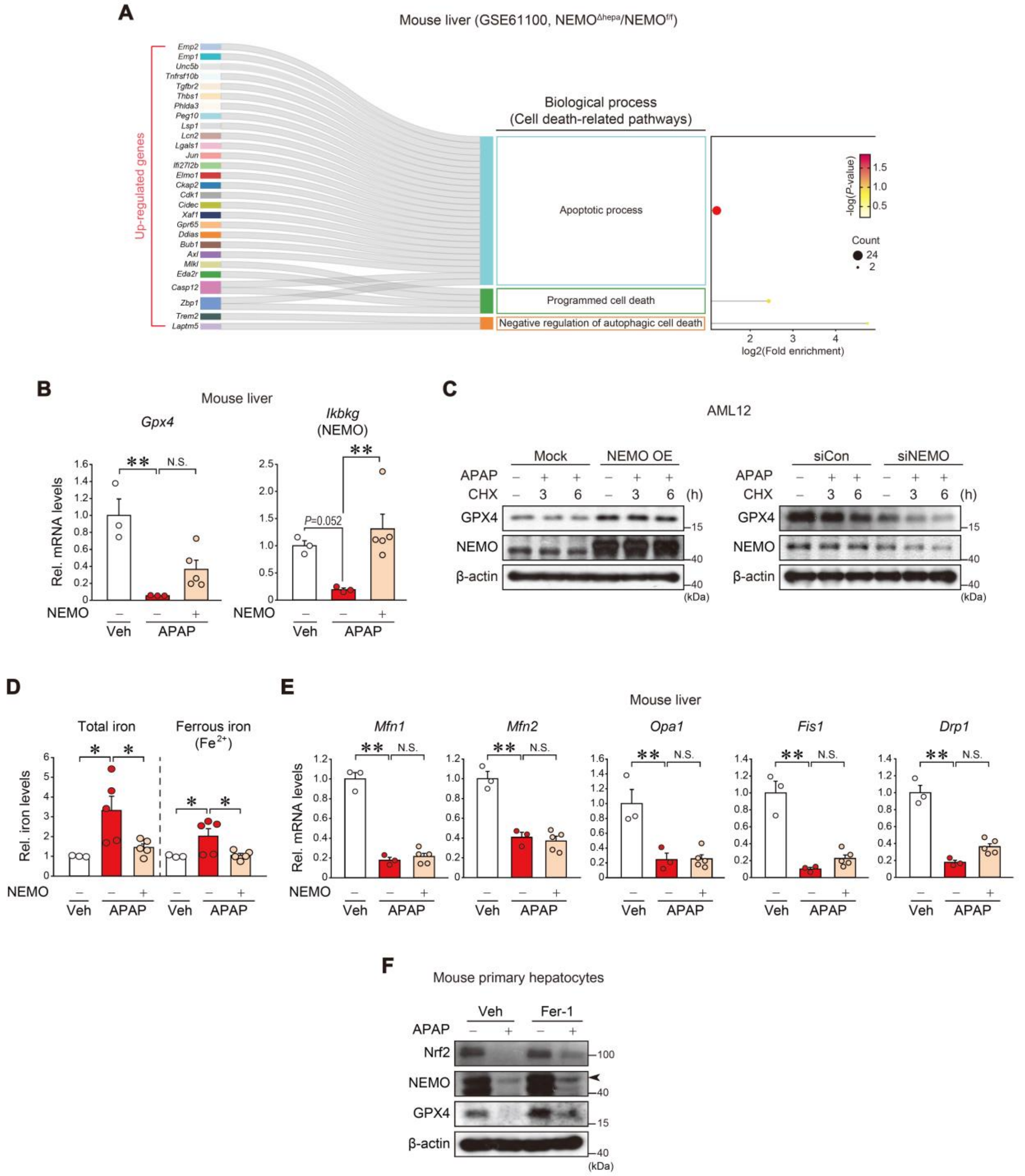

Figure S3. NEMO regulation of hepatocyte death, GPX4 stabilization, and ferrous iron content

(A) Cell death-related pathways based on the Sankey diagram (plot) of DEGs using hepatic transcriptome data from NEMO$^{f/f}$ or NEMO$^{\Delta hepa}$ mice. The Sankey diagram represents genes within each pathway; dot plots with sizes indicate gene numbers and dot colors display *P*-values (n = 3 each, DEGs of *P*-value < 0.01 and FC ≥ 2, GSE61100).

(B) qRT-PCR assays for *Gpx4* and *Ikbkg* in the same samples as in **Fig. 3C**.

(C) The effects of NEMO overexpression (OE) (left) or knockdown (right) on GPX4 stabilization. After transfection with NEMO OE or siNEMO for 24 h, AML12 cells were treated with APAP (12 h) and continuously exposed to cycloheximide (CHX) for the indicated times.

(D) Total and ferrous iron relative levels in the same samples as in **Fig. 3C**.

(E) qRT-PCR assays for mitochondrial fusion/fission genes in the same samples as in **Fig. 3C**.

(F) Immunoblottings for Nrf2, NEMO, and GPX4 in mouse primary hepatocytes treated with APAP (10 mM, 12 h) 1 h after vehicle or Fer-1 treatment (1 μM).

For **B**, **D**, and **E** values were expressed as mean ± SEM ($*P < 0.05$, $**P < 0.01$). Statistical significance was tested via one-way ANOVA coupled with Bonferroni's method or the LSD multiple comparison procedure when appropriate.

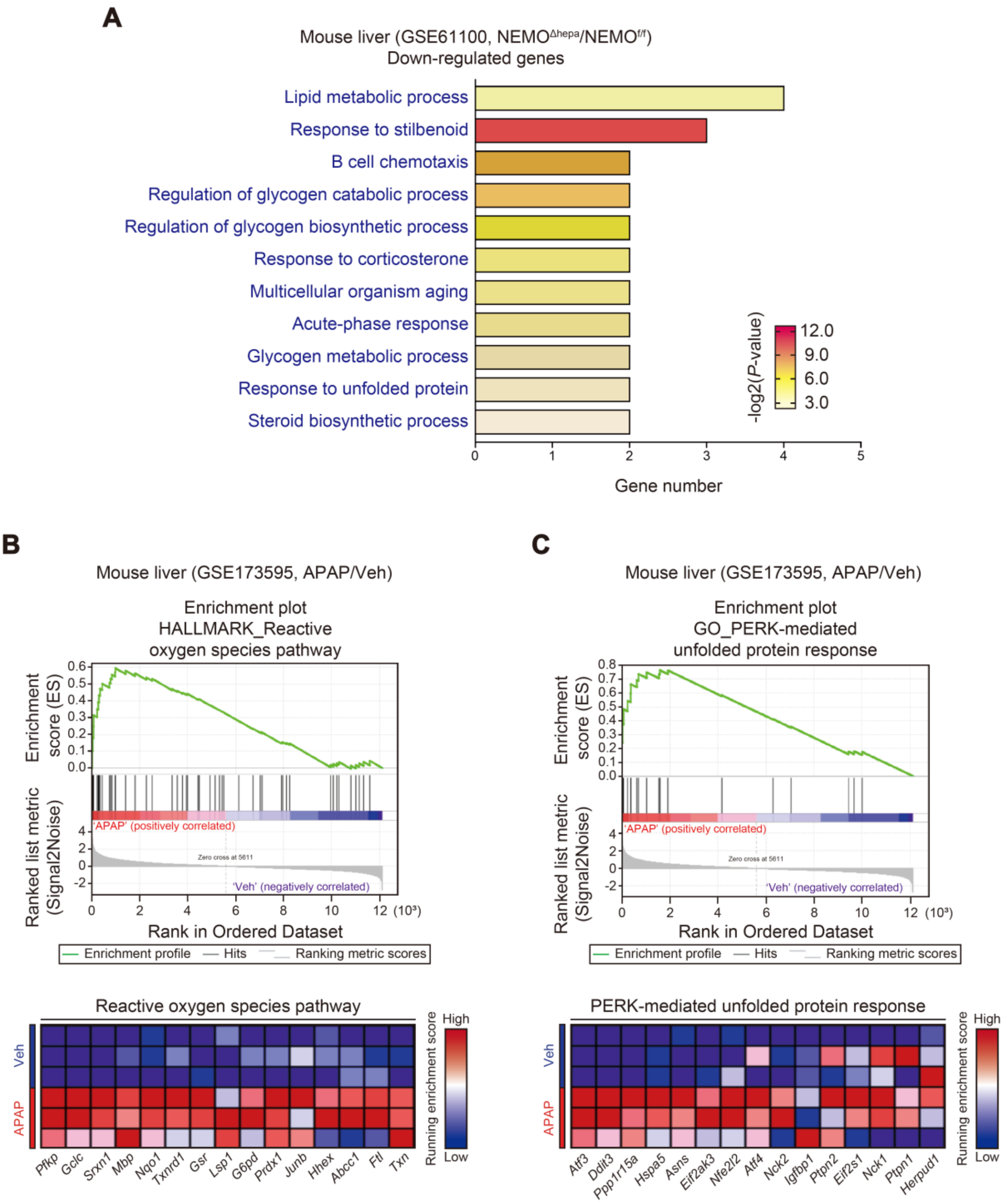

Figure S4. GO analysis of down-regulated genes by NEMO ablation in hepatocytes and APAP-induced ROS and ER stress pathways and the associated genes derived from hepatic transcriptome data in mice treated with APAP

(A) Gene ontology analysis of biological processes using hepatic transcriptome data from NEMO$^{f/f}$ or NEMO$^{\Delta hepa}$ mice (n = 3 each, DEGs of *P*-value < 0.01 and FC ≤ -2, GSE61100).

(B) GSEA enrichment plot of the hallmark showing the "Reactive oxygen species pathway" (upper). The top 15 genes comprising the leading edge of the enrichment score are shown in the corresponding heatmap (n = 3 each; blue, downregulation; red, upregulation; NES = 2.00, FDR < 0.0001) (lower).

(C) GSEA enrichment plot of the GO category showing the "PERK-mediated unfolded protein response" (upper). The top 15 genes comprising the leading edge of the enrichment score are shown in the corresponding heatmap (n = 3 each; blue, downregulation; red, upregulation; NES = 2.07, FDR < 0.0028) (lower).

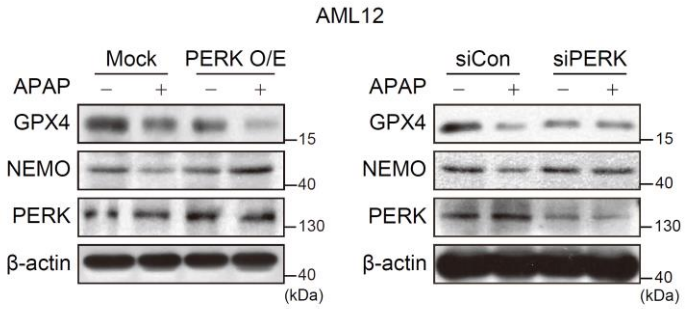

Figure S5. The effects of PERK modulations on GPX4 and NEMO levels

Immunoblottings for GPX4 and NEMO in AML12 cells treated with APAP (10 mM, 12 h) after transfection with PERK (or Mock) (1 µg, 24 h) (left) or siPERK (or siCon) (100 nM, 24 h) (right).

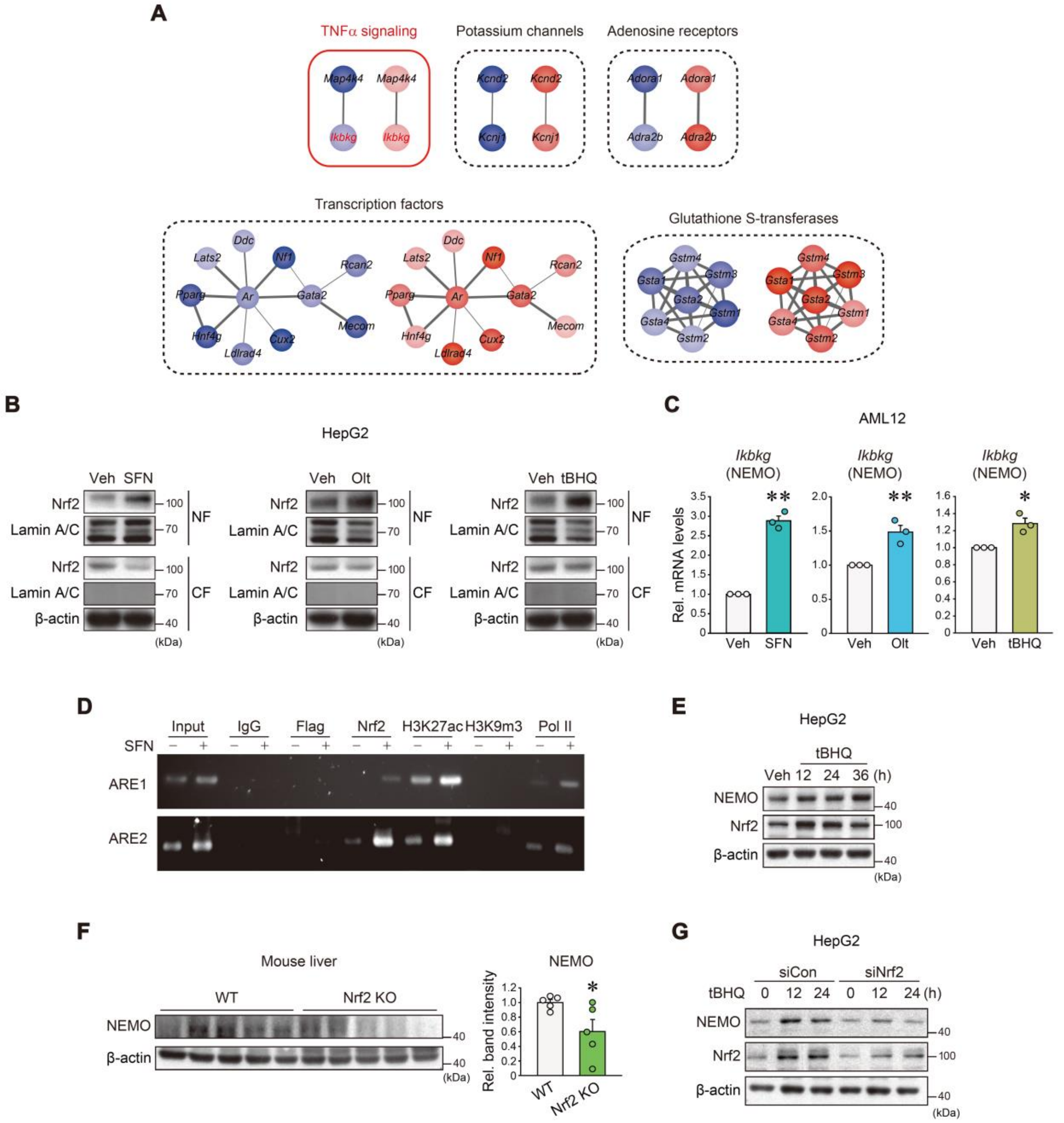

Figure S6. Nrf2 effect on NEMO gene expression

(A) The protein-protein interaction network of the genes, which are either decreased in Nrf2 knockout mice or increased in Keap1 knockout mice, was generated using the String database (GSE8969 and GSE11287, respectively).

(B) Immunoblottings for Nrf2 and Lamin A/C in the nuclear (NF) and cytoplasmic fractions (CF) of HepG2 cells treated with SFN (10 μM), Olt (30 μM), and tBHQ (30 μM) for 24 h.

(C) Real-time RT-PCR assays for *Ikbkg* in AML12 cells treated with SFN (10 μM), Olt (30 μM), and tBHQ (30 μM) for 24 h (n = 3).

(D) ChIP assays for Nrf2 binding to the putative antioxidant response element (ARE) regions in *IKBKG*. DNA-protein complexes were immuno-precipitated with Nrf2 in HepG2 cells treated with SFN for 24 h and then were subjected to PCR amplifications using the primers flanking the indicated AREs. IgG immunoprecipitation represents negative control. The specificity of Nrf2 binding was verified using the primers targeting an irrelevant region in the promoter. One-tenth of cross-linked lysates served as the input control.

(E) Immunoblottings for NEMO and Nrf2 in HepG2 cells treated with tBHQ (30 μM) for the indicated times.

(F) Immunoblottings for NEMO in the livers of WT and Nrf2 knockout mice (left). Band intensities represent values relative to each respective control (n = 5 each) (right).

(G) Immunoblottings for NEMO and Nrf2 in HepG2 cells treated with tBHQ (30 μM) after transfection with siNrf2 (or siCon) (100 nM, 24 h).

For **C and F**, values were expressed as mean ± SEM (**P* < 0.05, ***P* < 0.01). Statistical significance was tested via two-tailed Student's *t*-tests.

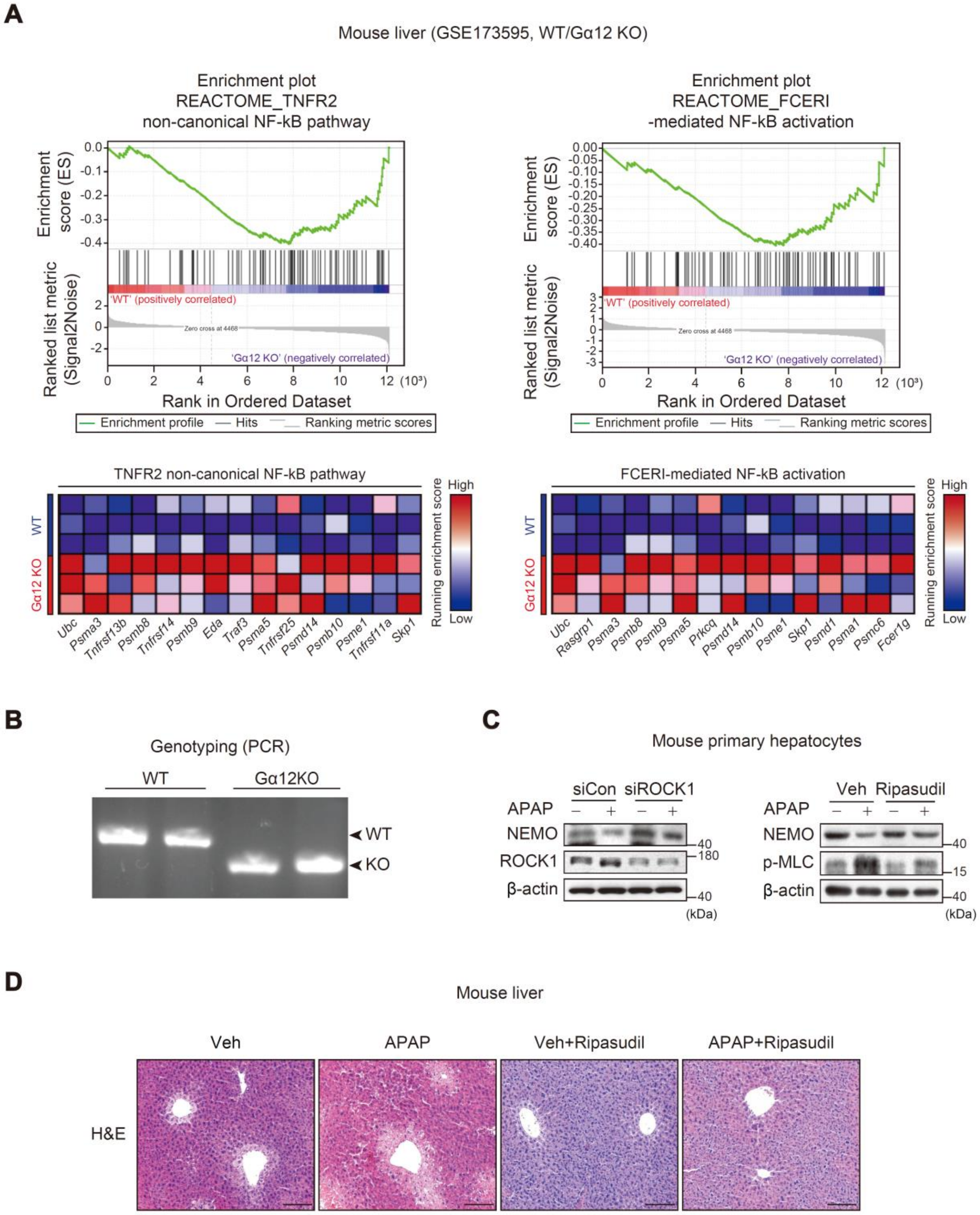

Figure S7. The roles of the Gα$_{12}$-ROCK1 axis in NF-κB-related pathways, NEMO levels, and APAP-induced histopathology

(A) GSEA enrichment plot of the Reactome showing the "TNFR2 non-canonical NF-κB pathway" and "FCERI-mediated NF-κB activation" derived from WT livers vs. $G\alpha_{12}$ KO livers (upper). The top 15 genes comprising the leading edge of enrichment score are shown in the corresponding heatmap (NES = -1.33, $P < 0.065$) (lower left) (NES = -1.32, $P < 0.073$, n = 3 each; blue, low; red, high) (lower right).

(B) PCR analysis for *Gna12* in genomic DNAs isolated from the tails of WT (littermates) and *Gna12* KO mice.

(C) Immunoblottings for NEMO, ROCK1, or p-MLC in mouse primary hepatocytes treated with APAP (10 mM, 12 h) after transfection with siROCK1 (siCon) (100 nM, 24 h) (left); or those similarly treated with APAP after treatment with ripasudil (50 μM, 1 h) (right).

(D) Liver histopathology in WT mice treated with ripasudil (50 mg/kg BW, 5 h) 1 h after vehicle (Veh) or APAP treatment (300 mg/kg BW, 6 h) (n = 3-5 each). Scale bar, 200 μm.

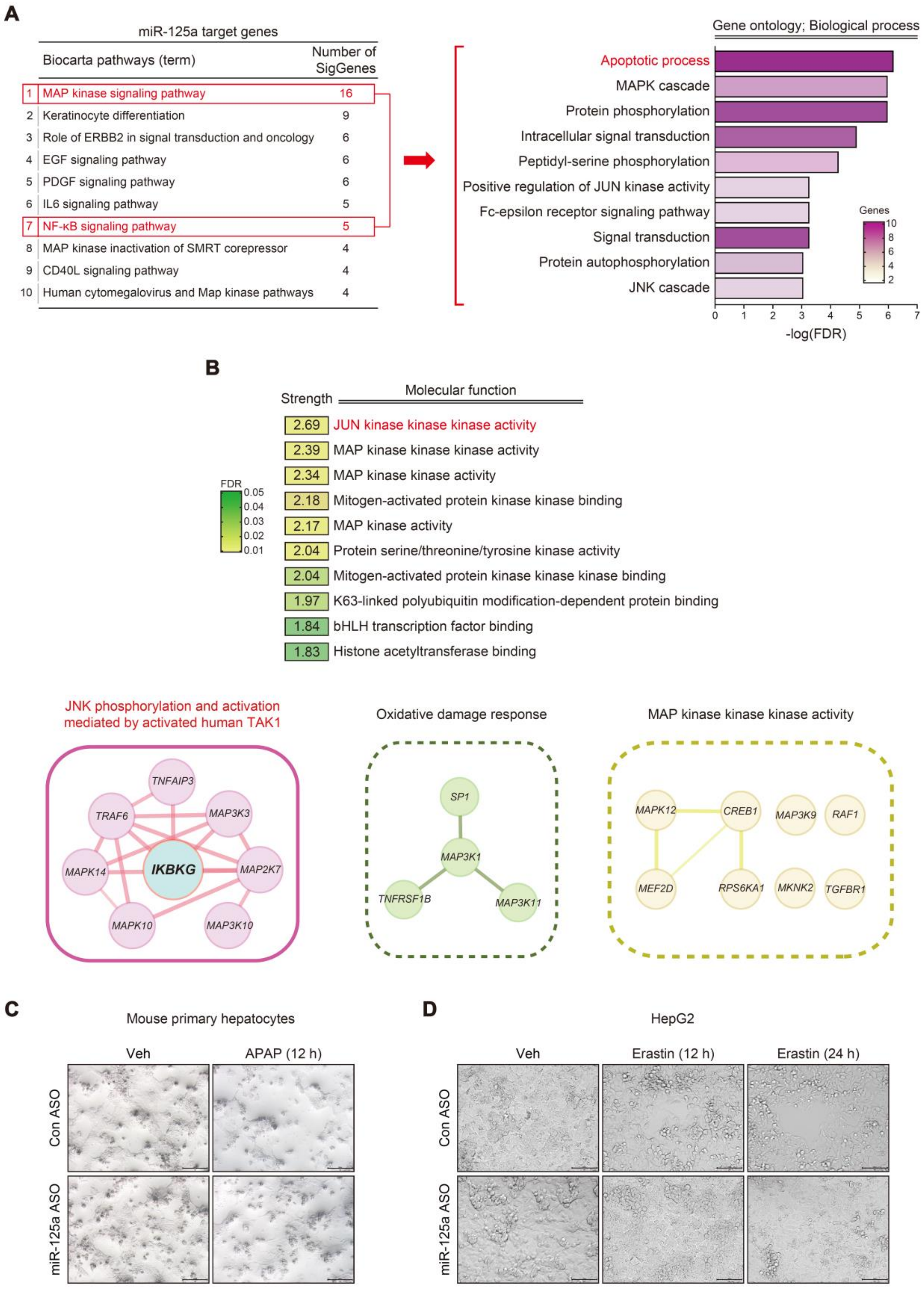
A
miR-125a target genes
Biocarta pathways (term)
Number of SigGenes
1 MAP kinase signaling pathway 16
2 Keratinocyte differentiation 9
3 Role of ERBB2 in signal transduction and oncology 6
4 EGF signaling pathway 6
5 PDGF signaling pathway 6
6 IL6 signaling pathway 5
7 NF-κB signaling pathway 5
8 MAP kinase inactivation of SMRT corepressor 4
9 CD40L signaling pathway 4
10 Human cytomegalovirus and Map kinase pathways 4
Gene ontology; Biological process
Apoptotic process
MAPK cascade
Protein phosphorylation
Intracellular signal transduction
Peptidyl-serine phosphorylation
Positive regulation of JUN kinase activity
Fc-epsilon receptor signaling pathway
Signal transduction
Protein autophosphorylation
JNK cascade
Genes
10
8
6
4
2
0 1 2 3 4 5 6 7
-log(FDR)
B
Strength
Molecular function
2.69 JUN kinase kinase kinase activity
2.39 MAP kinase kinase kinase activity
2.34 MAP kinase kinase activity
2.18 Mitogen-activated protein kinase kinase binding
2.17 MAP kinase activity
2.04 Protein serine/threonine/tyrosine kinase activity
2.04 Mitogen-activated protein kinase kinase kinase binding
1.97 K63-linked polyubiquitin modification-dependent protein binding
1.84 bHLH transcription factor binding
1.83 Histone acetyltransferase binding
FDR
0.05
0.04
0.03
0.02
0.01
JNK phosphorylation and activation mediated by activated human TAK1
TNFAIP3
TRAF6
MAP3K3
MAPK14
IKBKG
MAP2K7
MAPK10
MAP3K10
Oxidative damage response
SP1
MAP3K1
TNFRSF1B
MAP3K11
MAP kinase kinase kinase activity
MAPK12
CREB1
MAP3K9
RAF1
MEF2D
RPS6KA1
MKNK2
TGFBR1
C
Mouse primary hepatocytes
Veh
APAP (12 h)
Con ASO
miR-125a ASO
D
HepG2
Veh
Erastin (12 h)
Erastin (24 h)
Con ASO
miR-125a ASO

Figure S8. GO analysis using miR-125a target genes and morphology of mouse primary hepatocyte and HepG2 cells treated with APAP and Erastin after transfection with miR-125a ASO

(A) Gene ontology analysis using miR-125a target genes obtained from Targetscan. Prominent top 10 Biocarta pathways using the DAVID bioinformatics database (left). The GO term 'biological process' extracted with significant genes from MAP kinase and NF-κB pathways was associated with the apoptotic process (right).

(B) The GO term (molecular function) of the genes highlighted for functions such as JUN kinase kinase kinase activity (upper) and the protein-protein interaction network of the genes (lower), extracted with the number of significant genes from MAP kinase signaling pathway and NF-κB signaling pathway in the same GO analysis using miR-125a target genes as in Figure S8A left was generated using the String database.

(C) Representative morphology of mouse primary hepatocytes treated with APAP (12 h) after miR-125a ASO (or control ASO) transfection (100 nM, 48 h). Scale bar, 100 μm.

(D) Representative morphology of HepG2 cells treated with Erastin (5 μM, 12 or 24 h) after miR-125a ASO (or control ASO) transfection (100 nM, 48 h). Scale bar, 100 μm.

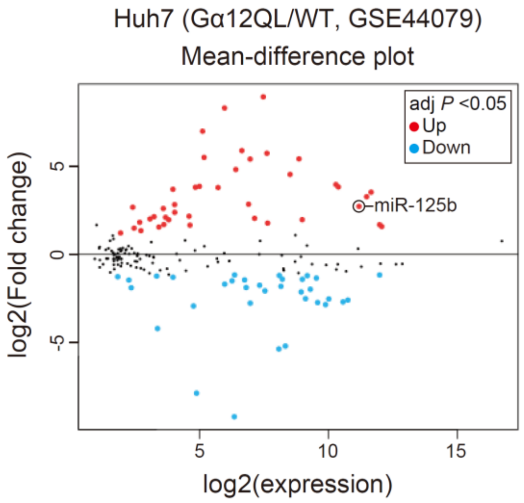

Figure S9. The effect of Gα_{12} modulations on a mean-difference plot of miRNA microarray

Red and blue dots show differentially expressed genes (DEGs) upregulated or downregulated by Gα_{12}QL (an active mutant form, n = 4 each).

Supplementary Table 1. The sequences of primers

Genes symbols (Human)	Forward	Reverse
IKBKG	TCATCGAGGTCCCATCAGGT	GAGACTCTTCGCCCAGTACG
CHUK	CAGCCATTTACCTGGCATGAG	GAGGGTCCCAATTCAACATCAA
IKBKB	ATCCCCGATAAGCCTGCCA	CTTGGGCTCTTGAAGGATACAG
GPX4	GAGGCAAGACCGAAGTAAACTAC	CCGAACTGGTTACACGGGAA
NFE2L2	CCTTCAGCAGCATCCTCTC	GCTTAAAGTAGCAGGTGAGGG
GAPDH	GAAGGTGAAGGTCGGAGTC	GAAGATGGTGATGGGATTTC
β-ACTIN	GATGAGATTGGCATGGCTTT	GTCACCTTCACCGTTCCAGT
**Genes symbols (Mouse)**	**Forward**	**Reverse**
Ikbkg	GAGGCCCTGGTAGCCAAAC	ATGGCAGCCAACTTTCAGCTT
Gpx4	TGTGCATCCCGCGATGATT	CACACGAAACCCTGTACTTATCC
Mfn1	ATGGCAGAAACGGTATCTCCA	GCCCTCAGTAACAAACTCCAGT
Mfn2	AGAACTGGACCCGGTTACCA	CACTTCGCTGATACCCCTGA
Opa1	TGGAATACAAAGAAACGTACCGC	GGGCAGGATGATGTGAACGA
Fis1	AGAGCACGCAATTTGAATATGCC	ATAGTCCCGCTGTTCCTCTTT
Drp1	CCTCAGATCGTCGTAGTGGGA	GTTCCTCTGGGAAGAAGGTCC
18S rRNA	GTAACCCGTTGAACCCCATT	CCATCCAATCGGTAGTAGCG
**microRNAs**	**Forward**	**Reverse**
miR-125a	TCCCTGAGACCCTTTAACCTGTGA	Universal reverse primer (Qiagen proprietary information)
miR-125b	TCCCTGAGACCCTAACTTGTGA	
miR-4319	TCCCTGAGCAAAGCCAC	
miR-365a	TAATGCCCCTAA AAATCCTTAT	
U6	GGGCAGGAAGAGGGCCTAT	
